# Genistein Exerts Neuroprotective Effects in an Ouabain-Induced Model of Bipolar Disorder: Behavioral and Molecular Insights

**DOI:** 10.1007/s11064-025-04597-3

**Published:** 2025-12-08

**Authors:** Mariam T. Arafat, Heba R. Ghaiad, Eman M. Elbaz

**Affiliations:** Department of Biochemistry, Faculty of Pharmacy, Cairo University, Kasr El Aini St. , Cairo, 11562 Egypt

**Keywords:** Bipolar disorder, Ouabain, Genistein, Na⁺/K⁺-ATPase signalosome, Mania, Depression

## Abstract

Bipolar disorder (BD) is a chronic and prevalent psychiatric disease that has been considered a leading cause of disability among psychiatric conditions. Taking into account that there is yet no satisfactory disease-modifying treatment, we investigated the effect of genistein on ouabain-induced BD in male C57BL/6 mice. Animals were categorized into control, genistein control, ouabain model, lithium (Li)-treated, and genistein-treated groups. BD was induced by bilateral intracerebroventricular injection of 0.625 nmol ouabain. Genistein (10 mg/kg/day) was orally administered for 2 weeks following a single dose of ouabain. Open field test, sucrose preference test, and forced swim test were performed. Na⁺/K⁺-ATPase activity was evaluated through measuring the hippocampal levels of phosphorylated epidermal growth factor receptor, proto-oncogene tyrosine-protein kinase, extracellular signal-regulated kinase, and cAMP response element-binding protein (p-CREB) by western blot analysis. The levels of brain-derived neurotrophic factor (BDNF), serotonin, oxidative stress, and inflammatory markers were quantified by ELISA. The BCL-2-associated X protein (BAX) to B-cell Lymphoma/Leukemia (BCL2) ratio was assessed by qRT-PCR. Genistein reduced manic and anxious behaviors during the manic phase and showed an antidepressant effect during the depression phase, all while maintaining an effective metabolic balance on body weight. Additionally, genistein increased serotonin, p-CREB, and BDNF levels while decreasing inflammation and apoptosis produced by ouabain. Furthermore, genistein restored the normal architecture in both hippocampal and cortical H&E-stained sections. Taken together, genistein was able to activate the Na⁺/K⁺-ATPase signalosome via a multifaceted mode of action, exerting a neuroprotective effect in an animal model of BD, promoting genistein as a therapeutic candidate for BD.

**Graphical Abstract:**

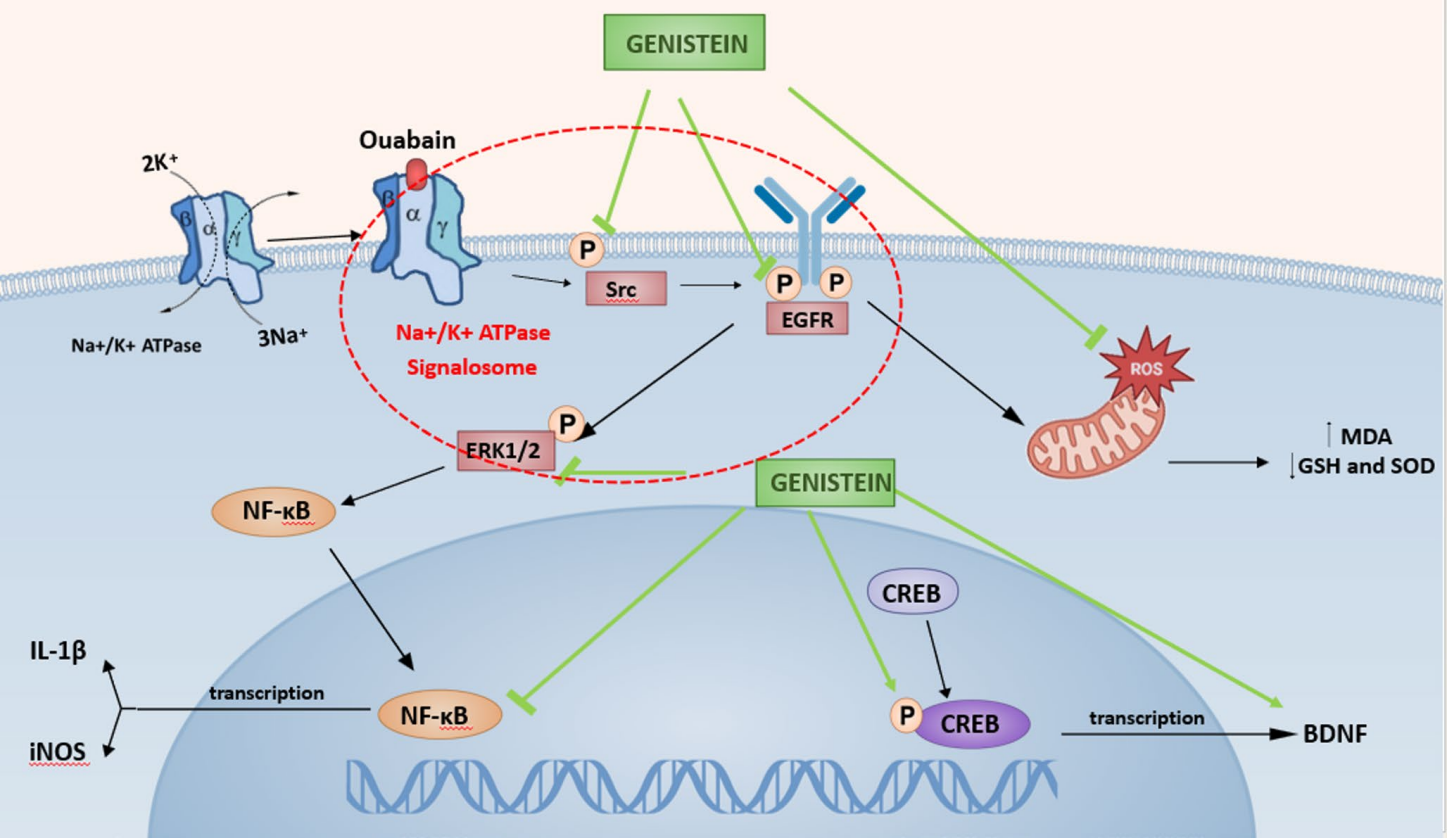

**Supplementary Information:**

The online version contains supplementary material available at 10.1007/s11064-025-04597-3.

## Introduction

 Bipolar disorder (BD) is an enduring and severe psychiatric disease, associated with elevated rates of lifelong psychiatric comorbidity [1]. BD is linked with derangements in numerous domains, including emotional, social, and cognitive functions [2]. It has been suggested that the hippocampus, amygdala, and their neural interconnections with the frontal cortex are fundamental in the pathophysiology of BD [3]. The distinctive hallmark of BD is the occurrence of acute manic episodes, which are characteristic of Bipolar I Disorder (BD I), while Bipolar II Disorder (BD II) is defined by the occurrence of hypomanic episodes and major depressive episodes [4].

The manic episodes of BD are characterized by psychomotor agitation, irritability, reduced need for sleep, and problematic behavior accompanied by impulsive involvement in high-risk activities. Conversely, the depressive phase is accompanied by sleep disturbances, including both insomnia and/or hypersomnia, in addition to anhedonia, fluctuations in body weight, and pervasive feelings of hopelessness. Between episodes, some BD patients have long periods of emotional stability. Others may frequently have mood swings from depression to mania or both depression and mania at the same time [5].

Mood-stabilizing treatments, mainly lithium (Li) and valproate, are used as primary therapeutic agents for managing acute mania, whereas Li remains to be the gold standard for the treatment of BD for over 50 years [6]. Owing to its narrow therapeutic index, Li intoxication is a prevalent issue among BD patients. Li toxicity can appear as neurological, gastrointestinal, cardiac, and renal manifestations in addition to nephrotoxicity and hepatotoxicity [7].

An indispensable tool to investigate BD is experimental animal models that enable screening drugs and studying the physiopathology of this disorder [8]. Ouabain is a cardiac glycoside that belongs to the family of digitalis steroids and has been demonstrated to induce symptoms comparable to those observed in individuals with BD when administrated via the intracerebroventricular (ICV) route to experimental animals [9]. The ouabain animal model of BD is connected with the suppression of the Na⁺/K⁺-ATPase in the brain, as such a pivotal enzyme has been previously implicated in mania in BD pathogenesis [10]. Na⁺/K⁺-ATPase is a transmembrane enzyme that is essential for preserving the electrochemical gradient within cells via keeping adequate levels of Na⁺, K⁺, and Ca²⁺ ions [11].

After binding, ouabain inhibits a series of protein–protein interactions, initiating numerous signaling pathways known as Na⁺/K⁺-ATPase signalosome [12]. Na⁺/K⁺-ATPase dysfunction has been implicated in the pathophysiology of psychiatric disorders by disrupting neuronal excitability and neurotransmitter balance, particularly within dopaminergic and glutamatergic pathways [13]. Additionally, Na⁺/K⁺-ATPase activity has been recently shown to be reduced in the brain and erythrocytes of BD individuals, which leads to heightened neuronal excitability, that could be a sufficient trigger to develop manic episodes in BD patients [14]. Imbalanced oxidative status is also a signature hallmark in ouabain-induced BD models, where ouabain-induced overproduction of malondialdehyde (MDA) has been demonstrated to cause alterations in major antioxidant defenses such as glutathione (GSH) and superoxide dismutase (SOD) [15].

Despite the devastating global impact of BD, its etiological and neurobiological mechanisms remain largely elusive. Multiple intermingled cellular pathways have been postulated to be implicated in BD, including dysregulations in the metabolism and action of cholinergic, glutaminergic, GABAergic, serotonin, and opioid neurotransmission [16]. Furthermore, strong evidence showed that both inflammation and apoptosis contribute to BD etiology [17]. In addition, the universal transcription factor, nuclear factor-kB (NF-κB), has participated in neuroinflammatory status triggered by ouabain [18].

The usage of nutraceuticals in the psychiatric field has been representing a promising approach that recently attracted the attention of researchers for their therapeutic effects against several illnesses, including psychiatric disorders like BD [19]. In fact, pure flavonoids have the potential to penetrate the blood brain barrier (BBB) with minimal harmful consequences [20]. Previously, genistein was reported to demonstrate a cognitive-improving impact via controlling NF-κB-mediated brain-derived neurotrophic factor (BDNF) expression [21].

BDNF is amongst the extensively studied neurotrophic factors in psychiatric disorders, as it has been demonstrated to support neuronal maturation, differentiation, and survival [22]. Moreover, BDNF contributes essentially to the activity of cAMP-response element binding protein (CREB), that modulates long-term synaptic potentiation, survival, neurogenesis, and finally neuronal plasticity [23]. Perturbed BDNF and CREB within the hippocampus have been previously associated with memory loss and cognitive dysfunction [24].

Owing to the neuroprotective, anti-oxidative, and anti-inflammatory characteristics, genistein has been used to restore and enhance memory in various experimental animal models and in humans [25]. However, no attempt has been made to determine whether genistein could reverse the neuronal impairment induced by ouabain till now.

Therefore, the current study was directed to scrutinize the putative neuroprotective effects of genistein against ouabain-triggered behavioral abnormalities, biochemical derangements, and histological changes in mice. The study also aimed to elaborate on the impact of genistein on the pathogenetic pathway involving modulating Na⁺/K⁺-ATPase signalosomes, downregulating oxidative stress, cytokine-induced signal transduction events, and focusing on its important anti-inflammatory, anxiolytic, and antidepressant effects on the ouabain mouse model of BD.

## Materials and Methods

### Animals

Male C57BL/6 mice (9–10 weeks old; 25–30 g) were purchased from the Theodor Bilharz Research Institute (Cairo, Egypt). Animals were housed 5 per cage in accordance with “Mouse Cage Density IACUC Policy − 2020” in polycarbonate cages with corn cobs bedding under controlled conditions of temperature and humidity, with a12 hour light/dark alternating cycle all over the study. Mice were given unlimited access to water and a typical rodent chow. Animals were permitted to adapt 7 days before the study [26]. Animals handling, in addition to all the experimental techniques, were completed in accordance with the guide for the care and use of laboratory animals and were permitted by the Ethics Committee for Animal Experimentation at the Faculty of Pharmacy, Cairo University (Permit Number: BC 3591). Every necessary endeavor was undertaken to lessen animal agony and decrease the overall number of experimental animals utilized. Additionally, all experimental procedures, housing conditions, and handling techniques were optimized to reduce pain, distress, and suffering of the mice.

### Drugs and Chemicals

Ouabain (Cat. No.: 1076) and artificial cerebrospinal fluid (aCSF, Cat. No.: 3525) were purchased from Tocris Bioscience (UK). Genistein (Cat. No.: HY-N0595) was supplied by MedChem Express (USA). Li carbonate was obtained from Sanofi (Egypt), and sodium salt of carboxy methyl cellulose (CMC-Na) was obtained from Teba Chemical Industry (Egypt). All other chemicals and reagents were of the highest purity.

### Induction of Bipolar Disease

Prior to induction of BD, an intraperitoneal (i.p.) injection of 5 mg/kg thiopental was used to anesthetize mice [27]. A single bilateral ICV injection of 0.625 nmol ouabain dissolved in 2.5 µL aCSF [28] was inserted into each lateral ventricle of the brain[29] according to the freehand procedure. The needle was inserted unilaterally at an equal distance between the eyes and the ears and perpendicular to the plane of the skull [30].

### Experimental Design

**Fig. 1:**
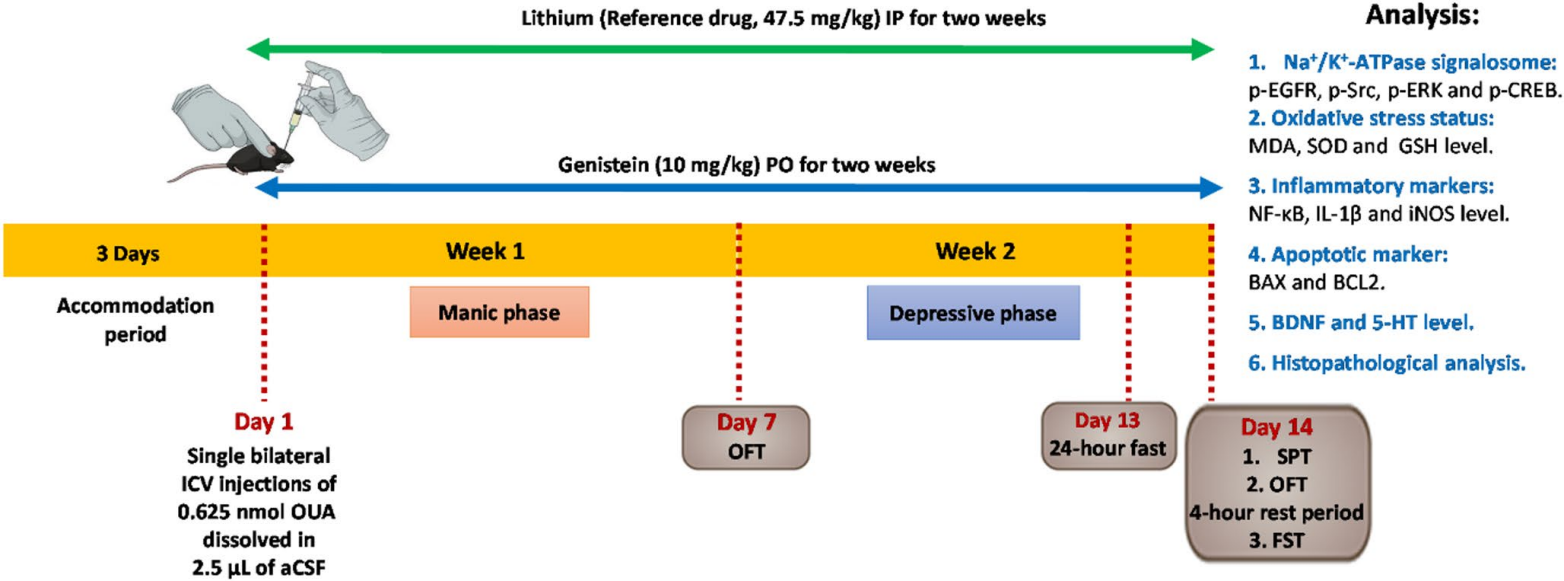
Timeline of the experimental design. 5-HT: 5-hydroxytryptamine or serotonin; aCSF: artificial cerebrospinal fluid; BAX: BCL-2-associated X protein; BCL2: B-cell Lymphoma/Leukemia; BDNF: brain-derived neurotrophic factor; FST: Forced swim test; GSH: glutathione; ICV: intracerebroventricular; IL-1β: interleukein-1β; iNOS: inducible nitric oxide synthase; IP: intraperitoneal; MDA: malondialdehyde; NF-κB: nuclear factor kappa B; OFT: Open field test; OUA: ouabain; PO: per os, or by mouth; p-CREB: phosphorylated cAMP response element-binding protein; p-EGFR: phosphorylated epidermal growth factor receptor; p-ERK: phosphorylated extracellular signal-regulated kinase; p-Src: phosphorylated proto-oncogene tyrosine-protein kinase; SOD: superoxide dismutase; SPT: Sucrose preference test

Sixty male mice were split up randomly into 5 groups. Group 1: normal control group (*n* = 12), where mice received a single ICV injection of 2.5 µL of aCSF bilaterally in addition to 0.5% CMC-Na administered orally for 2 weeks to represent the control vehicle group and evaluate baseline values of various parameters. Group 2: genistein control group (*n* = 12), received a single ICV injection of 2.5 µL of aCSF bilaterally in addition to 10 mg/kg/day genistein dissolved in 0.5% CMC-Na given orally for 2 weeks [31]. Group 3: Ouabain group (*n* = 12), where mice received a single ICV injection of 0.625 nmol ouabain dissolved in 2.5 µL of aCSF into each lateral ventricle to serve as the model [29]. Group 4: Li-treated group (*n* = 12), where mice received ICV injection of ouabain as in group 3, in addition to 47.5 mg/kg Li carbonate dissolved in phosphate-buffered saline (PBS) that is administered i.p. twice daily for 2 weeks [32]. Group 5: Genistein-treated group (*n* = 12), mice received ICV injection of ouabain as in group 3, in addition to 10 mg/kg/day genistein dissolved in 0.5% CMC-Na and delivered orally for 2 weeks[31]. The timeline of the experimental process was summarized in (Fig. 1).

Animal body weights were recorded weekly. On the 7th day, animals were subjected to the open field test (OFT). On the 14th day, behavioral testing was conducted in a sequential order from the least to the most strenuous: first, the sucrose preference test (SPT), followed by a second OFT, and finally the forced swim test (FST). Afterwards, animals were sacrificed, and each experimental group was divided into two subsets; the first set (*n* = 3) brains were separated and fixed in 10% formalin for histopathological examination. In the second set (*n* = 9), the two hippocampi from each brain were dissected and frozen at − 80 °C for biochemical analysis. Animals were disposed of according to the Faculty of Pharmacy, Cairo University (FOPCU) guidelines of animal handling and disposal. During the analysis of all samples, the identity of the sample was hidden from the investigator, where a separate investigator performed the sample coding and decoding.

### Neurobehavioral Assessment

The neurobehavioral assessments were performed sequentially from the least to most stressful at room temperature in a calm room without any outside interferences. Each experiment was carried out while the animal was maintained under a 12:12 h light/dark cycle to diminish any circadian-related variance.

#### Sucrose Preference Test (SPT)

After a 24-hour fast, SPT was carried out early in the morning of the fourteenth day of the experiment to evaluate anhedonia. SPT was carried out according to the previously approved method [33]. In brief, mice were not given water and food for a full day, then housed in cages with full accessibility to two bottles: one containing 100 ml of water and the other having 100 ml of 1% sucrose solution (w/v). After 24 h, the consumed volumes from both bottles were documented, and sucrose preference was assessed via the following equation:

$$\begin{aligned}&\mathrm{Sucrose}\:\mathrm{preference}\:(\%)\\&=\left[\frac{sucrose\:intake}{water\:intake\:+\:sucrose\:intake}\right]\times\:100\end{aligned}$$

The SPT relies on the animal’s inherent inclination towards sweets, suggesting that this tendency correlates with the pleasure derived from getting sweetened solutions, a method commonly employed to evaluate an animal’s sensitivity to reward, referred to as hedonia. Decreased sucrose consumption reflects anhedonia; this has been recognized as a well-validated indicator of a depressive-like state in animals [34], homologous to that of depressed humans [35].

#### Open Field Test (OFT)

OFT was executed on the 7th and 14th days to assess motor activity and anxious behavior in mice and evaluate the willingness of rodents to explore new environments [35]. In this test, the mice were placed on a wooden white floor box (40 cm × 60 cm) enclosed by 50 cm wood walls. The floor is divided into identical squares separated by black lines. The open field was cleaned each time after testing a mouse, even if it was clean before the first trial. It is crucially important to clean the open field box so that any remaining smell of the disinfectant is experienced similarly by all mice to minimize possible olfactory animal bias [36]. Four squares were taken as the “center” of the field. For five minutes, every mouse was carefully placed in the left rear quadrant to explore the surroundings. The locomotor and exploratory activities were assessed as mean speed, mobility time, distance travelled, time spent in the center, and number of rearing. Short periods spent in the middle of the open field and low levels of mobile activity were frequently seen as signs of high anxiety, and vice versa [9]. During the test, the activity of the experimental animals was monitored using a video camera positioned above the open field in a uniformly lit room. Behavioral data were photographed, recorded, and analyzed using the ANY-maze video tracking system (ANY-maze, USA).

#### Forced Swim Test (FST)

To assess passive and depressive-like behavior, FST was conducted once on the 14th day of the experiment, following the completion of the OFT and after giving a 4-hour rest period for the mice. This test was a derivation of Porsolt et al.’s method [37]. In order to minimize differences and irregularities in the immobility duration across groups, we briefly put the mice through a “pretest session” [38]. Mice were forced to swim individually in glass cylindrical tanks (19 × 12 cm) containing water up to a height of 10 cm at a temperature of 25 °C, which was enough for the mice to swim freely without touching the bottom with their paws or tail. The session lasted for a total period of 6 min in which the first 2 min were considered as a “pretest session” and the last 4 min were the “test session”. The time of immobility was then recorded in the preceding 4 min of each session [39]. Mice were considered immobile if they floated still in the water, making only movement necessary to maintain their heads above the water. Reduction in the length of immobility time was considered an antidepressant effect [40]. The swimming behavior of the animals was recorded using a video camera positioned laterally to the cylinder and analyzed using the ANY-maze video tracking system (ANY-maze, USA).

### Biochemical Investigations

Under mild anesthesia, using 5 mg/kg thiopental i.p. the animals were euthanized via cervical dislocation [41]. Afterwards, hippocampi were quickly separated, cleaned with cold PBS, dried, and weighed.

#### Western Blot Analysis

Western blot analysis was carried out as earlier defined [42]. In 1 ml TriFast (Cat. No. 30–2010, Peqlab, UK), 50–100 mg of hippocampal tissues have been homogenized on ice via CellLytic MT mammalian tissue lysis reagent (Cat. No. C3228, Sigma-Aldrich, USA) containing protease and phosphatase inhibitor cocktails (Cat. No. PPC1010, Sigma-Aldrich, USA). The homogenates were denatured at 100 °C for 5 min and then centrifuged at 10,000 g for 30 min. Electrophoresed proteins on SDS-PAGE, which is sodium dodecyl sulfate–polyacrylamide gel electrophoresis, were transferred to BA85 nitrocellulose membrane (Ref. 401–491, Schleicher and Schuell, Germany) and incubated for one hour at room temperature in blocking solution.

For the western blot analysis, primary antibodies were used at room temperature including anti p-ERK 1/2 (Cat. No. sc-81492, Santa Cruz Biotechnology, USA) at a dilution of (1/1000–1/10000), anti p-CREB (Cat. No. mAb #9196, Cell Signaling Technology, USA) at a dilution of 1/1000, anti p-EGFR (Cat. No. ab40815, Abcam, UK) at a dilution of (1/1000–1/10000), and anti p-Src (Cat. No. ab4816, Abcam, UK) at a dilution of 1/1000. and anti β-Actin antibody (Cat. No. ab8226, Abcam, UK) at a dilution of 1/1000. β-actin was applied to be the housekeeping protein. After washing, the membranes were incubated with a secondary antibody containing peroxidase-conjugated anti-rabbit immunoglobulin G (IgG) (Cat. No. NA934V, GE Healthcare, USA) or anti-mouse IgG (0.1–0.5 µg/ml, Cat. No. 7076, Cell Signaling Technology, USA) for one hour at room temperature. Antibody concentrations were adjusted between 0.05 and 2.0 µg/ml to obtain the desired signal strength and low background. Gel documentation system was applied for data analysis using Totallab analysis software (Ver.1.0.1). As a loading control, β-actin was used to standardize band intensities.

The protein fraction concentration was determined according to the Bradford (1976) method based on the interaction between protein and Coomassie Brilliant Blue G250 (CBBG-250) in acidic conditions. An amount of 50 µl of distilled water and 200 µl Coomassie Blue Reagent was added to 50 µl of protein extract. After color stabilization for 5 min, the absorbance at 595 nm was recorded. Protein sample concentrations were determined in reference to a series of standards based on bovin serum albumin (BSA).

#### Enzyme Linked Immunosorbent Assay (ELISA) Assays

Hippocampal tissues were homogenized in ice-cold PBS, then the homogenates were used for the assay of oxidative stress parameters using a mouse GSH ELISA kit (Cat. No. MBS267424, MyBioSource, USA), a mouse MDA ELISA kit (Cat. No. MBS741034, MyBioSource, USA), and a mouse SOD ELISA kit (Cat. No. MBS2707323, MyBioSource, USA) according to manufacturer procedures. Hippocampal tissue concentrations of GSH, MDA, and SOD were expressed as ng/mg protein.

The Bradford technique was employed to determine the protein concentration of various subcellular fractions [43]. Protein Assay Kit (Cat. No. 23200, Thermo Fisher Scientific, USA). Neuroinflammatory parameters were estimated using a mouse ELISA Kit for NF-κB (Cat No. MBS2023542, MyBioSource, USA), mouse iNOS (Cat No. MBS261100, MyBioSource, USA), and mouse IL-1β (Cat No. ab197742, Abcam, UK). Hippocampal NF-κB concentrations were reported as ng/mg protein, and iNOS was expressed as U/mg protein, whereas IL-1β levels were expressed as pg/mg protein. Hippocampal homogenates were also used for the assay of BDNF using the ELISA Kit (Cat No. MBS355435, MyBioSource, USA) and serotonin using the 5-HT ELISA kit (Cat No. E-EL-0033, Elabscience, USA). BDNF was expressed as pg/mg protein, and serotonin was expressed as ng/mg protein.

#### Gene Expression Analysis (qRT-PCR)

Quantitative reverse transcriptase polymerase chain reaction (qRT-PCR) was employed to measure the gene expression of the apoptotic markers BAX and BCL2. Total RNA was extracted from tissue lysate with Direct-zol RNA Miniprep Plus (Cat No. R2072, ZYMO Research Corp., USA) then the quantity and quality of the extracted RNA were assessed by a Beckman dual spectrophotometer (USA). The optical density (OD) of extracted RNA was measured at 260 nm and 280 nm to ensure RNA purity. SuperScript IV One-Step RT-PCR kit (Cat No. 12594100, Thermo Fisher Scientific, USA) was utilized for reverse transcription of extracted RNA, followed by qPCR in one step. Table 1 provides the primer sequences that were used. The thermal cycler protocol was 10 min at 55 °C, 2 min at 95 °C, 40 cycles of 10 s at 95 °C for denaturation, 10 s at 55 °C for annealing, and 30 s at 72 °C for extension. Glyceraldehyde 3-phosphate dehydrogenase (GAPDH), a housekeeping gene, was used to correct the variations in target gene expression, which was then subsequently determined using the ΔΔCT method and reported as fold change (FC = 2-ΔΔCT) [44].

**Table 1:** Primer sequences for each gene examined

	Forward sequence (5’→3’)	Reverse sequence (3’→ 5’)
BAX	CTACAGGGGTGAGTGCGATG	TTCTTGGTGGACGCATCCTG
BCL2	ACTTTCCATGGACGCGTTTG	CTCCGCAATGCTGAAAGGTG
GAPDH	GGGGATGCCATAAGGAGGAG	AGCACTCTCCCCACCTCAAT

#### Histopathological Examination

Specimens of hippocampi were fixed in phosphate buffered formalin 10% (PBF), pH = 7.4, and processed by the paraffin embedding technique. Transverse sections of 4–5 μm thickness were prepared and dyed with hematoxylin and eosin (H & E) [45], then blindly examined under a light microscope by a pathologist (BX43, Olympus Life Science, USA). Additionally, histopathological scoring was assessed in brain tissue for the quantity of deteriorated neurons per cross-sectional area utilizing the Leica QWin DW3000 image processing system (LEICA Imaging Systems Ltd., Cambridge, England). For every segment in every group, the six most representative fields were evaluated using light microscopy relayed to the screen at 400x magnification [46].

#### Statistical Analysis

Statistical evaluations were carried out by GraphPad Prism - version 10.2.3 (GraphPad Software, USA). The Shapiro–Wilk test was used to test the normality of the studied datasets. One-way analysis of variance (ANOVA) followed by Holm-Šídák’s multiple comparisons test was used for normally distributed data. Non-parametric data were analyzed by Kruskal-Wallis analysis of variances followed by Dunn’s multiple comparisons test. When comparing the means of two independent groups, the Mann-Whitney U-test was used for data that was not parametric, while the unpaired two-tailed t-test was utilized for parametric data. The results were presented as mean ± standard error (SEM). *P* < 0.05 was considered to be statistically significant.

## Results

### Effect of Genistein on Body Weight in Ouabain-Induced BP in Mice

**Fig. 2:**
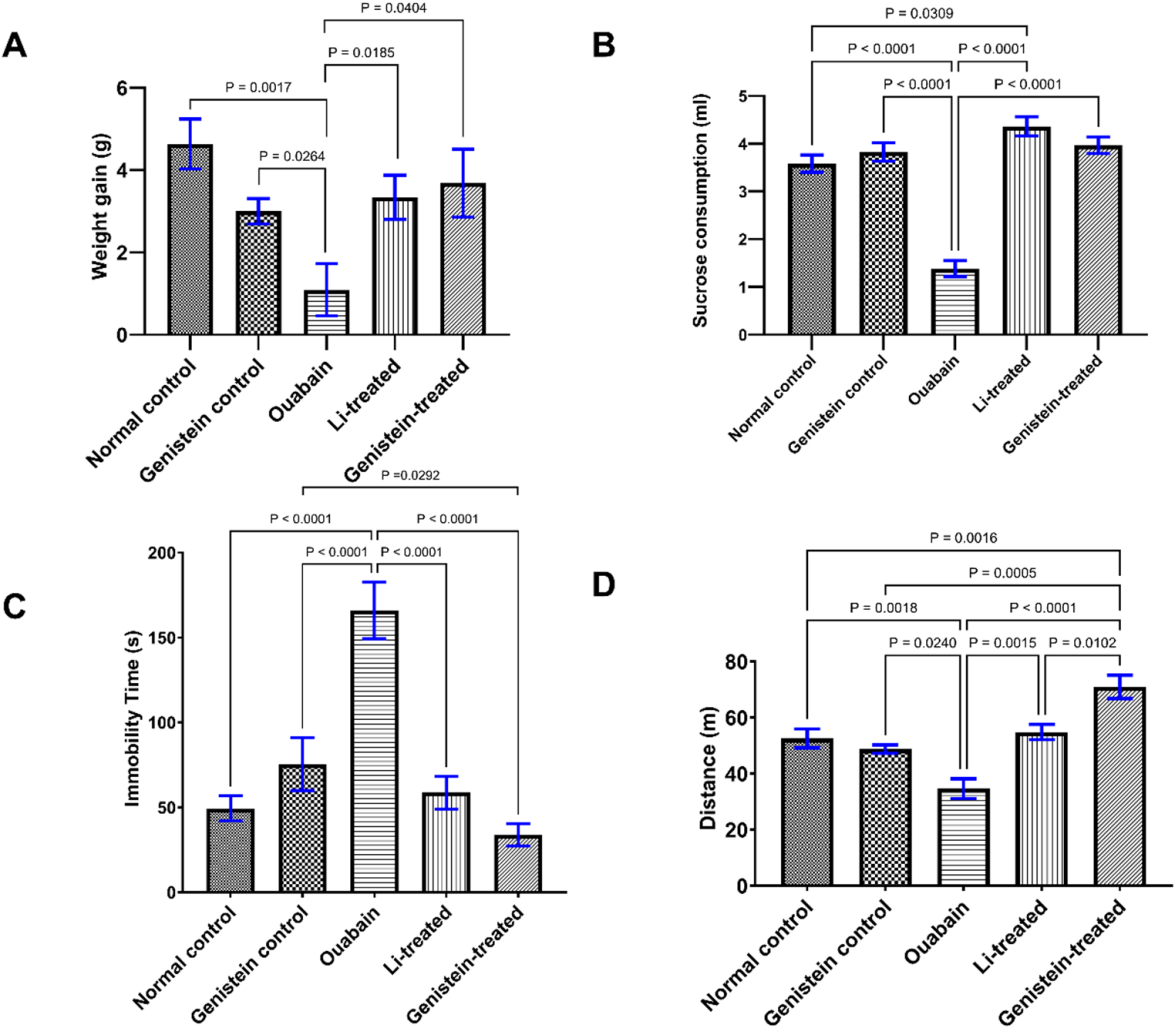
Effect of genistein on (A) body weight, (B) sucrose consumption in sucrose preference test, and (C and D) immobility time and distance in forced swim test in ouabain-induced BD in mice. Differences among groups were analyzed by Kruskal-Wallis test in (A), while statistical differences were analyzed by ordinary one-way ANOVA test in (B, C and D). Each column represents the mean of 7–10 mice ± SEM. Statistical significance was considered at *P* < 0.05

As shown in (Fig. 2A), a single ICV administration of ouabain to C57BL/6 mice caused a significant weight loss over 2 weeks in comparison to normal control animals (*P* = 0.0017). Treatment with genistein significantly amended the decline in body weight induced by ouabain (*P* = 0.0404). Treatment with Li also caused a significant increase in weight gain, returning it to normal control values (*P* = 0.0185) in comparison with the ouabain group values. Both the impacts of genistein and Li seemed comparable and not statistically significant from one another (*P* < 0.9999).

### Effect of Genistein on Sucrose Consumption in Ouabain-Induced BD in Mice

Fourteen days after ICV administration of ouabain, there was a significant decrease in sucrose consumption to half that of normal control values (Fig. 2B). Treatment with genistein was successful in normalizing sucrose consumption (*P* < 0.0001, Fig. 2B) in contrast to the ouabain group. Treatment with the standard drug, Li also caused a marked improvement in sucrose consumption when compared to the ouabain group (*P* < 0.0001, Fig. 2B). Both the effects of genistein and Li were comparable to one another (*P* = 0.4451, Fig. 2B).

### Effect of Genistein on Immobility time and Distance Travelled in the Forced Swim Test in Ouabain-Induced BD in Mice

Fourteen days after ICV administration of ouabain, immobility time in the forced swim test was significantly increased (*P* < 0.0001, Fig. 2C), while distance travelled was significantly decreased (*P* = 0.0018, Fig. 2D) in contrast to control animals. Genistein treatment successfully reversed all the changes in behavior caused by ouabain administration, as it showed a significant decrease in immobility time (*P* < 0.0001, Fig. 2C) in conjugation with a significant increase in distance travelled (*P* < 0.0001, Fig. 2D) when compared to the ouabain group. Similarly, treatment with Li demonstrated a significant diminish in immobility time (*P* < 0.0001, Fig. 2C) and a substantial rise in distance travelled (*P* = 0.0015, Fig. 2D) when compared to the ouabain group. The effects of genistein and Li on immobility time (*P* = 0.6410, Fig. 2C) were not statistically significant from one another, whereas the effects of genistein on distance travelled were superior to that of Li (*P* = 0.0102, Fig. 2D).

### Effect of Genistein on the Activity of Mice in the Open Field Test in Ouabain-Induced BD

The OFT was performed at the end of each phase of ouabain-induced BD in mice; the 1 st time it was performed on day 7 after ICV administration of ouabain to demonstrate the manic phase, then repeated on day 14 to analyze the behavioral changes in the depressive phase.

- **Open field test performed 7 days after ouabain injection**
  Ouabain administration increased the mean speed (*P* = 0.0473, Fig. 3A), mobility time (*P* = 0.0214, Fig. 3B), distance (*P* = 0.0038, Fig. 3C), centre time (*P* = 0.0155, Fig. 3D), and number of rearing (*P* = 0.0005, Fig. 3E) in C57BL/6 mice when compared to normal controls. Genistein treatment reversed all the behavioural changes inflicted by ouabain administration, which was shown by a decrease in the mean speed (*P* = 0.0446, Fig. 3A), mobility time (*P* = 0.0214, Fig. 3B), distance (*P* = 0.0111, Fig. 3C), centre time (*P* = 0.0284, Fig. 3D), and number of rearing (*P* = 0.0019, Fig. 3E) as compared to the ouabain group. Furthermore, Li treatment caused a significant decrease in the mean speed (*P* = 0.0466, Fig. 3A), mobility time (*P* < 0.0001, Fig. 3B), distance (*P* = 0.0175, Fig. 3C) and number of rearing (*P* = 0.0041, Fig. 3E) when compared to the ouabain group values. However, the effect of Li in centre time was not statistically significant from the ouabain group values (*P* = 0.2426, Fig. 3D). Both the effects of genistein and Li on mean speed, distance travelled, and number of rearing were comparable and statistically non-significant from one another (Fig. 3A, C and E respectively), whereas genistein was superior to Li in increasing mobility time (*P* = 0.0224, Fig. 3B).
  Moreover, track plots and heat maps of mice during the OFT confirmed increased locomotor activity in the ouabain group compared to normal control animals, and normalized activities in both genistein and Li-treated groups (Fig. 3F and G).
  **Fig. 3:**
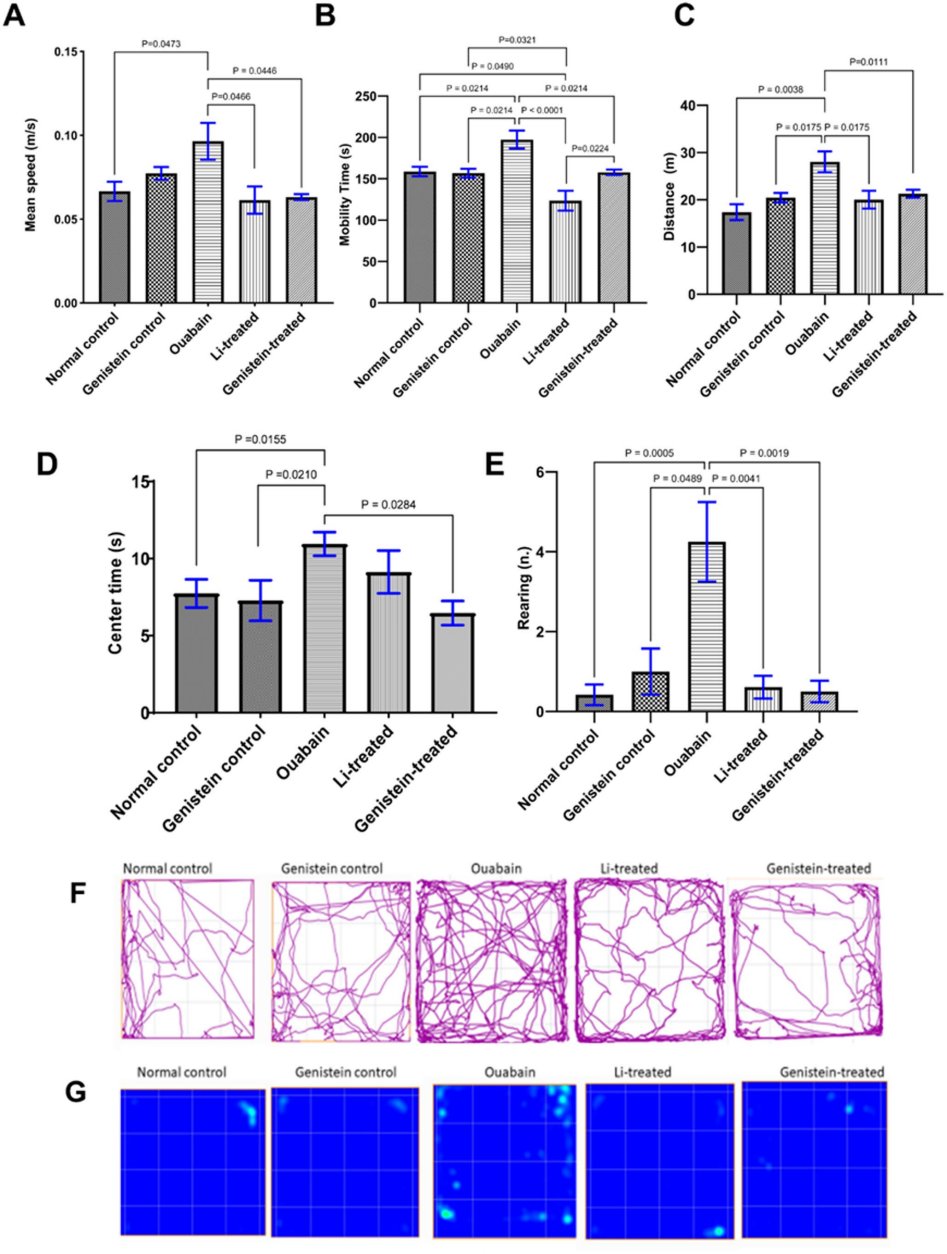
Effect of genistein on the activity of mice in the open field test in ouabain-induced BD after 7 days from ouabain injection. (A, B and C) show the effect of genistein on motor activity per 5 min in the open field, where (A) Mean speed, (B) Mobility time, and (C) Distance travelled. (D and E) show the effect of genistein on anxious behavior, where (D) Centre time, (E) Number of rearing, (F) Track plots and (G) Heat maps of mice during the OFT. Variations between groups were examined by Kruskal-Wallis test in (A, C and E), while the evaluation of statistical variations was done by the ordinary one-way ANOVA test in (B and D). The relevance was evaluated at *P* < 0.05. Each column represents the mean of 7–10 mice ± SEM
- **Open field test performed 14 days after ouabain injection**.
  On the 14th day after ouabain injection, ouabain group showed a considerable decrease in the mean speed (*P* = 0.0001, Fig. 4A), mobility time (*P* < 0.0001, Fig. 4B), distance (*P* = 0.0230, Fig. 4C), center time (*P* < 0.0001, Fig. 4D) and number of rearing (*P* < 0.0001, Fig. 4E) when compared to normal controls. Genistein treatment ameliorated all behavioral alterations induced by ouabain administration, as evidenced by a reduction in the mean speed (*P* = 0.0006, Fig. 4A), mobility time (*P* = 0.0010, Fig. 4B), distance (*P* = 0.0115, Fig. 4C), centre time (*P* = 0.0010, Fig. 4D) and decreased the frequency of rearing (*P* = 0.0473, Fig. 4E) as compared to the ouabain group. Similarly, treatment with the standard drug, Li abolished all of the behavioral alterations that were caused by the administration of ouabain, as demonstrated by an increase in the mean speed (*P* = 0.0313, Fig. 4A) and centre time (*P* = 0.0011, Fig. 4D) and decreased the number of rearing (*P* = 0.0015, Fig. 4E) as compared to the ouabain group. However, the effects of Li on mobility time (*P* = 0.1891, Fig. 4B) and distance (*P* > 0.9999, Fig. 4C) were not statistically significant from the ouabain group values. The effects of genistein and Li on mean speed and centre time were statistically non-significant from one another (Fig. 4A, C and D, and E). Meanwhile, genistein was superior to Li in increasing mobility time (*P* = 0.0435, Fig. 4B).
  Moreover, track plots and heat maps of mice during the OFT confirmed decreased locomotor activity in the ouabain group compared to normal control animals, and normalized activities in both genistein and Li-treated groups (Fig. 4F and G).
  **Fig. 4:**
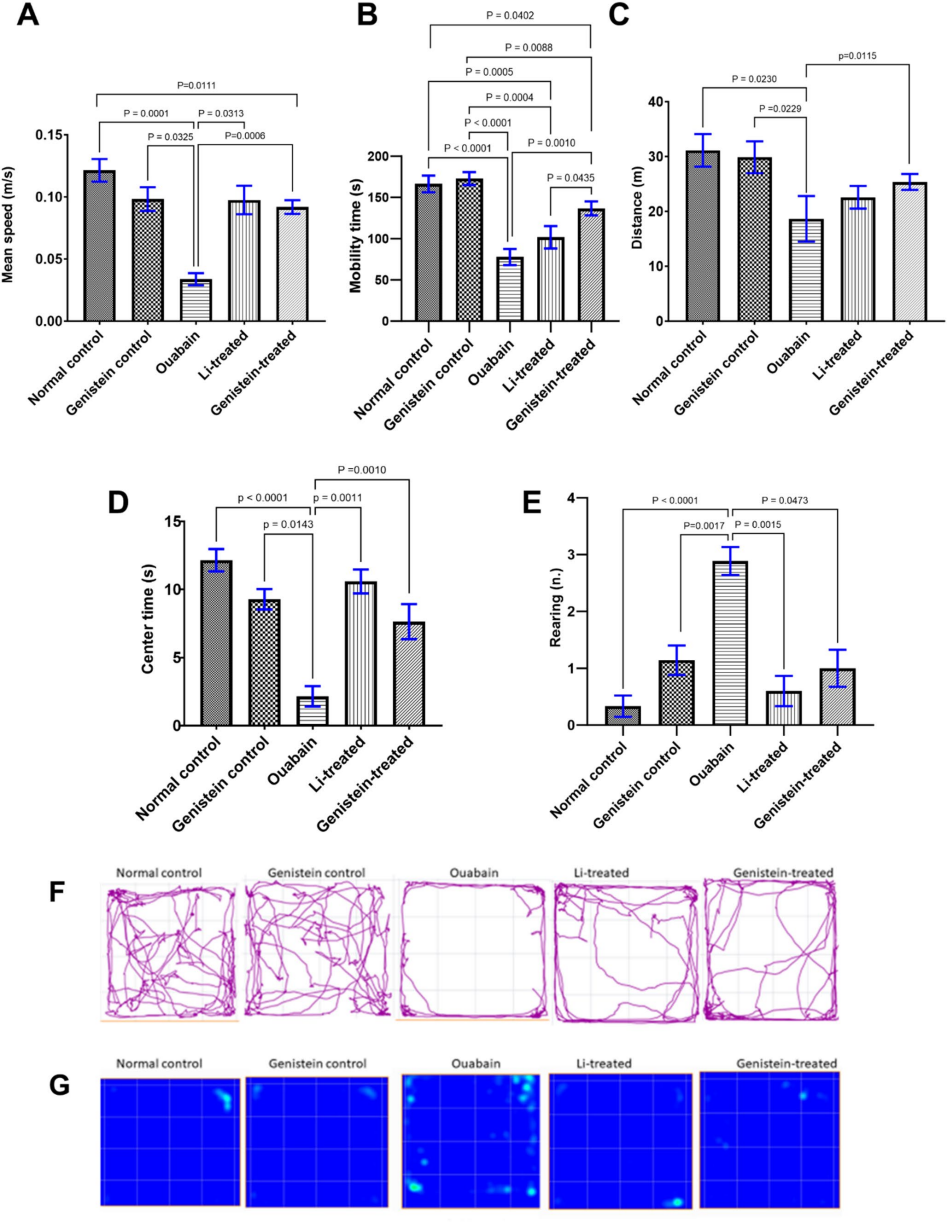
Effect of genistein on the activity of mice in the open field test in ouabain-induced BD 14 days after ouabain injection. (A, B and C) show the effect of genistein on motor activity per 5 min in the open field, where (A) Mean speed, (B) Mobility time, and (C) Distance travelled. (D and E) show the effect of genistein on anxious behavior, where (D) Centre time (E) Rearing time, (F) Track plots, and (G) Heat maps of mice during the OFT. Differences among groups were analyzed by Kruskal-Wallis test in (A, C, D and E), where the statistical variation has been investigated via the ordinary one-way ANOVA test in (B). The statistical significance was considered at *P* < 0.05. Each column represents the mean of 7–10 mice ± SEM

### Effect of Genistein on Activation Na⁺/K⁺-ATPase Signalosome in Ouabain-Induced BD in Mice

**Fig. 5:**
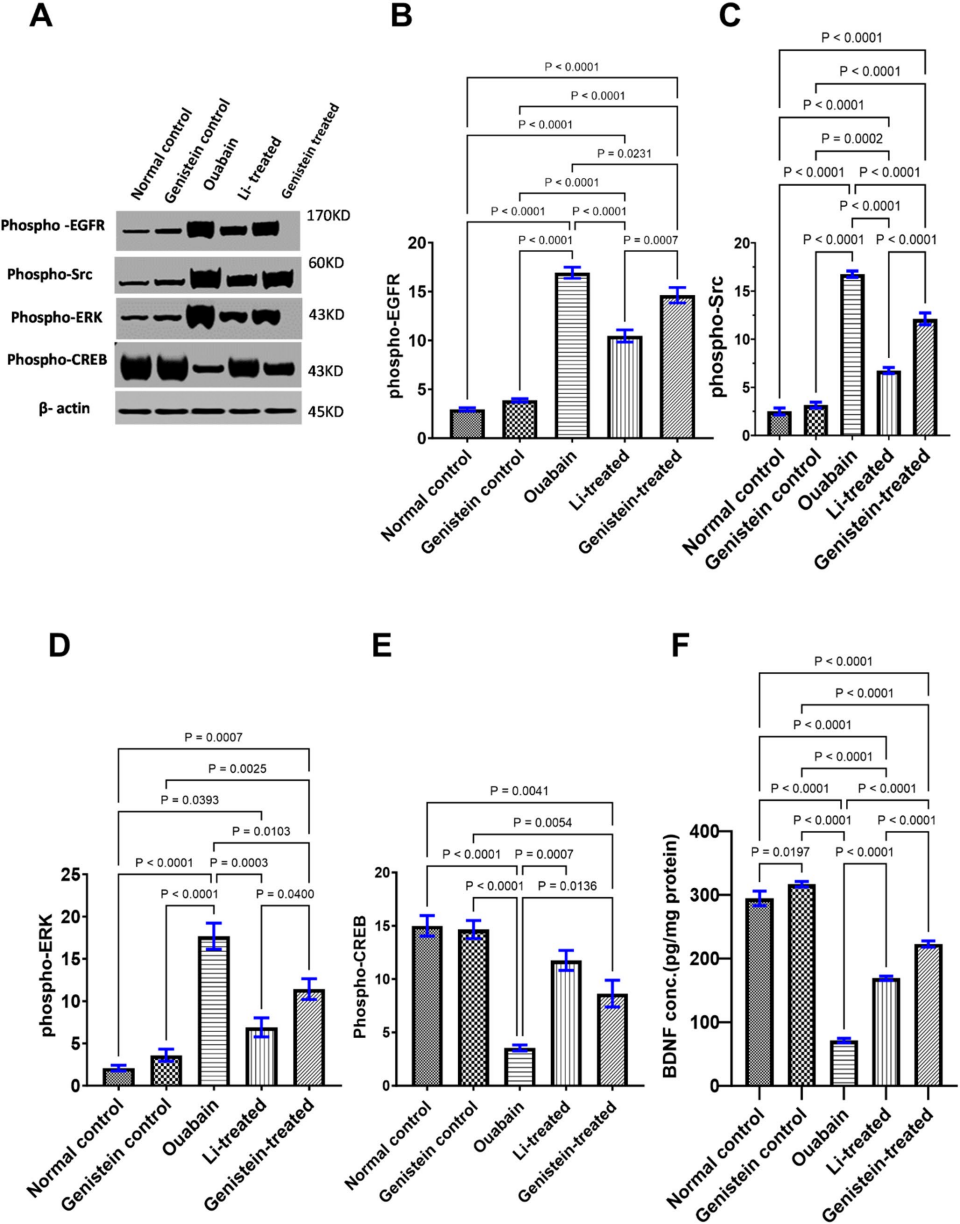
Effect of genistein on the activation of hippocampal Na⁺/K⁺-ATPase signalosome in ouabain-induced BD in mice. (A) Representative photographs showing the protein expression of the phospho-EGFR, phospho-Src, phospho-ERK and phospho-CREB in relation to β-actin as a reference protein (B) phospho-EGFR level, (C) phospho-Src level, (D) phospho-ERK level, (E) phospho-CREB level, and (F) BDNF level. Group differences have been evaluated by the ordinary one-way ANOVA test. Each column represents the mean of 3 mice ± SEM, except in BDNF *n* = 6. Statistical relevance was considered at *P* < 0.05

In comparison to the normal group, ouabain group showed an increase in phospho-EGFR, (*P* < 0.0001, Fig. 5B), phospho-Src (*P* < 0.0001, Fig. 5C), and phospho-ERK (*P* < 0.0001, Fig. 5D). Furthermore, ouabain caused a marked decline in phospho-CREB (*P* < 0.0001, Fig. 5E), subsequently a substantial decrease in BDNF levels (*P* < 0.0001, Fig. 5F) in the hippocampus in comparison to normal control values. On the contrary, genistein administration reversed the effects inflicted by ouabain injection, evidenced by a prominent reduction in phospho-EGFR (*P* = 0.0231, Fig. 5B), phospho-Src (*P* < 0.0001, Fig. 5C), and phospho-ERK protein (*P* = 0.0103, Fig. 5D) when compared to the ouabain group. Genistein caused a marked increase in phospho-CREB (*P* = 0.0136, Fig. 5E) and in BDNF level (*P* < 0.0001, Fig. 5F) in the hippocampus when compared to the ouabain group.

Likewise, treatment with Li reversed all the changes that ouabain injection caused, indicated by a significant reduction in phospho-EGFR (*P* < 0.0001, Fig. 5B), phospho-Src (*P* < 0.0001, Fig. 5C), and phospho-ERK (*P* = 0.0003, Fig. 5D) when compared to the ouabain group. Moreover, Li caused a marked increase in phospho-CREB (*P* = 0.0007, Fig. 5E) and BDNF level (*P* < 0.0001, Fig. 5F) in the hippocampus compared to the ouabain group.

Effects of Li were superior to genistein in decreasing phospho-EGFR (*P* = 0.0007, Fig. 5B), phospho-Src (*P* < 0.0001, Fig. 5C) and phospho-ERK (*P* < 0.0400, Fig. 5D). Nevertheless, the effects of genistein on BDNF protein levels were superior to that of Li (*P* < 0.0001, Fig. 5F), whereas, in terms of p-CREB, both Li and genistein were comparable to each other.

### Effect of Genistein on Oxidative Stress Status in Ouabain-Induced BD in Mice

**Fig. 6:**
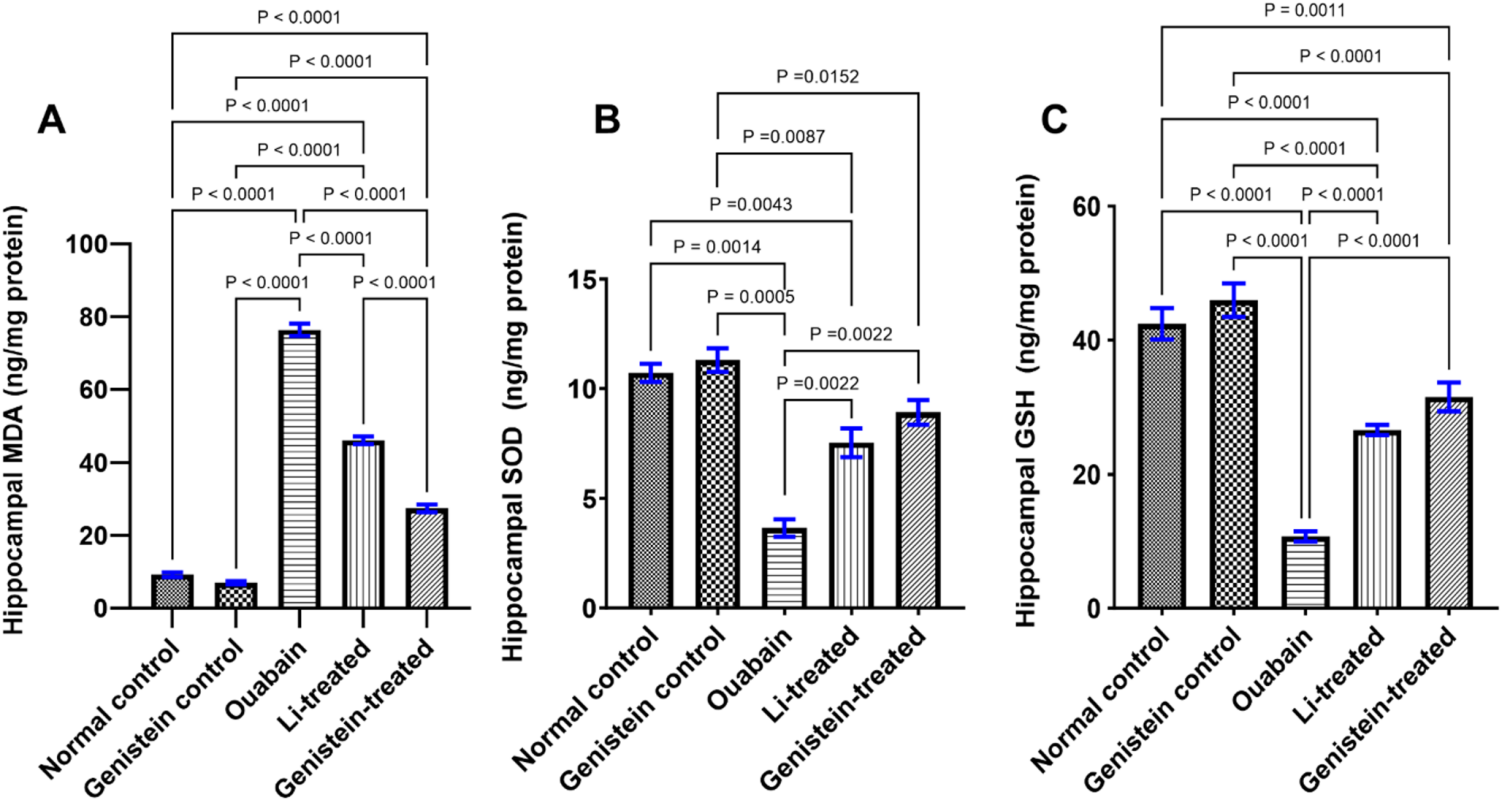
Effect of genistein on hippocampal oxidative stress status in ouabain-induced BD in mice. (A) MDA level, (B) SOD level, and (C) GSH level. Differences among groups were analyzed by ordinary one-way ANOVA test in (A and C), while statistical relevancies were analyzed by Kruskal-Wallis test in (B). Each column represents the mean of 6 mice ± SEM. Statistical significance was considered at *P* < 0.05

The current findings indisputably show major modifications in the oxidative stress markers in the ouabain group’s hippocampal tissue. This was illustrated by a notable increase in MDA level (*P* < 0.0001, Fig. 6A) and a substantial decrease in SOD activity (*P* = 0.0014, Fig. 6B) and GSH level (*P* < 0.0001, Fig. 6C) in the hippocampus when compared to a normal control group. Genistein treatment successfully corrected the oxidative stress state abnormalities caused by ouabain, as evidenced by a notable decrease in MDA level (*P* < 0.0001, Fig. 6A) and increased both SOD activity (*P* = 0.0022, Fig. 6B) and GSH level (*P* < 0.0001, Fig. 6C) in the hippocampus when compared to the ouabain group. Similarly, the administration of Li effectively repaired the imbalances in oxidative stress status produced by ouabain, illustrated as a significant reduction in MDA level (*P* < 0.0001, Fig. 6A) and increase both SOD activity (*P* = 0.0022, Fig. 6B) and GSH level (*P* < 0.0001, Fig. 6C) in the hippocampus when compared to the ouabain group. Both the effects of genistein and Li treatments on SOD and GSH levels were not statistically significant from one another (Fig. 6B and C). Whereas the effects of genistein on MDA level were superior to that of Li (*P* < 0.0001, Fig. 6A).

### Effect of Genistein on Inflammatory Markers in Ouabain-Induced BD in Mice

**Fig. 7:**
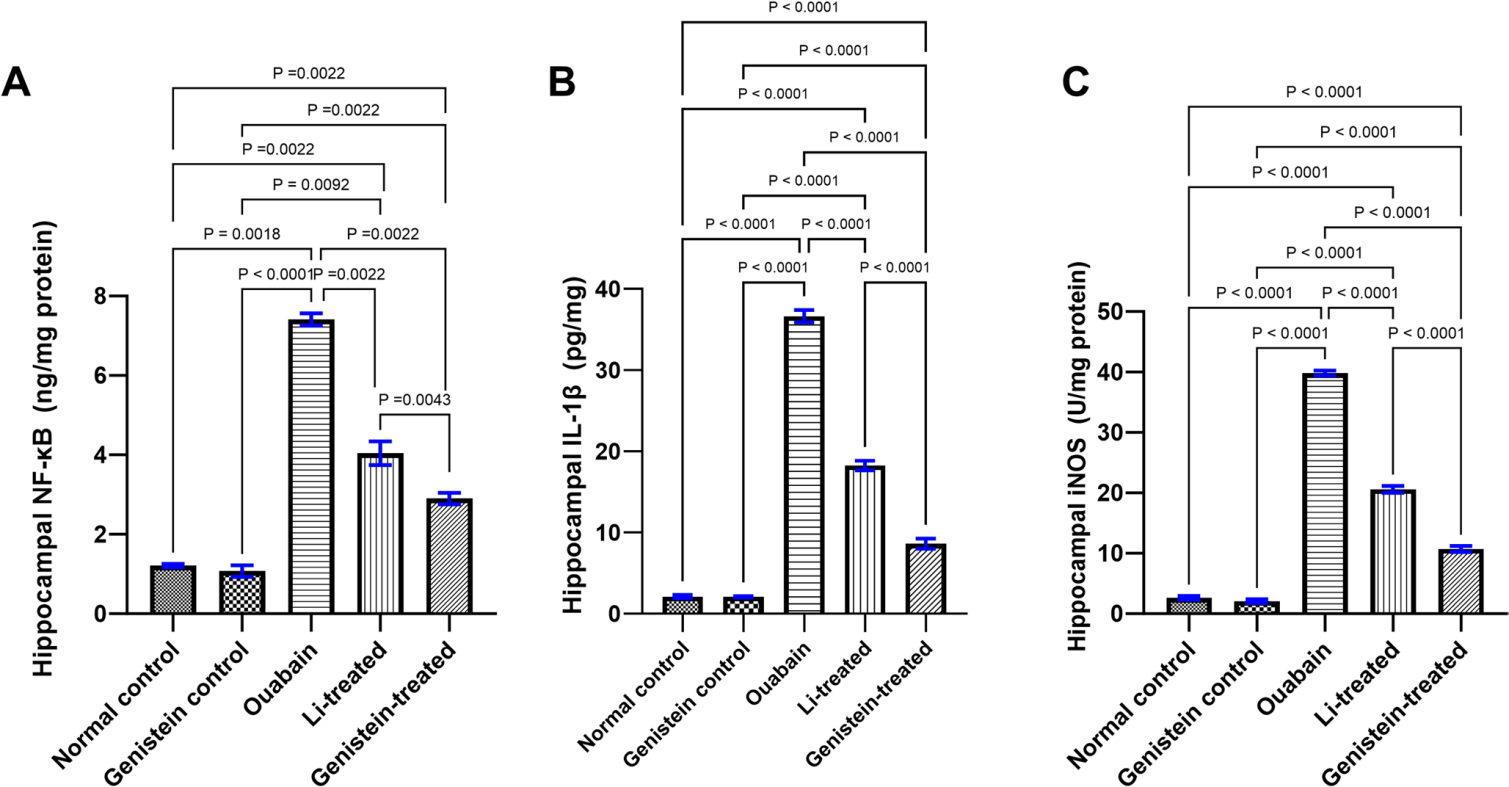
Effect of genistein on hippocampal inflammatory markers in ouabain-induced BD in mice. (A) NF-κB level, (B) IL-1β level and (C) iNOS level. Differences among groups were analyzed by Kruskal-Wallis test in (A), while the statistical differences were analyzed by ordinary one-way ANOVA test in (B and C). Each column represented the mean of 6 mice ± SEM. Statistically significant difference was considered at *P* < 0.05

Ouabain increased NF-κB level (*P* = 0.0018, Fig. 7A), in addition to the pro-inflammatory cytokines, such as IL-1β (*P* < 0.0001, Fig. 7B) and iNOS level (*P* < 0.0001, Fig. 7C) in the hippocampus in comparison to the normal control group. When genistein was administered, the inflammatory markers alterations induced by ouabain were significantly reversed demonstrated by decreased NF-κB expression (*P* = 0.0022, Fig. 7A) as well as IL-1β (*P* < 0.0001, Fig. 7B) and iNOS level (*P* < 0.0001, Fig. 7C) in the hippocampus when compared to the ouabain group. Furthermore, treatment with the standard drug, Li reversed all of the inflammatory alterations caused by ouabain, causing a significant reduction in NF-κB level (*P* = 0.0022, Fig. 7A) and IL-1β (*P* < 0.0001, Fig. 7B), and iNOS concentrations (*P* < 0.0001, Fig. 7C) in the hippocampus when compared to ouabain-induced group. The effects of genistein on NF-κB level (*P* = 0.0043, Fig. 7A), IL-1β (*P* < 0.0001, Fig. 7B) and iNOS level (*P* < 0.0001, Fig. 7C) were superior to those of Li.

### Effect of Genistein on Apoptotic Markers in Ouabain-Induced BD in Mice

**Fig. 8:**
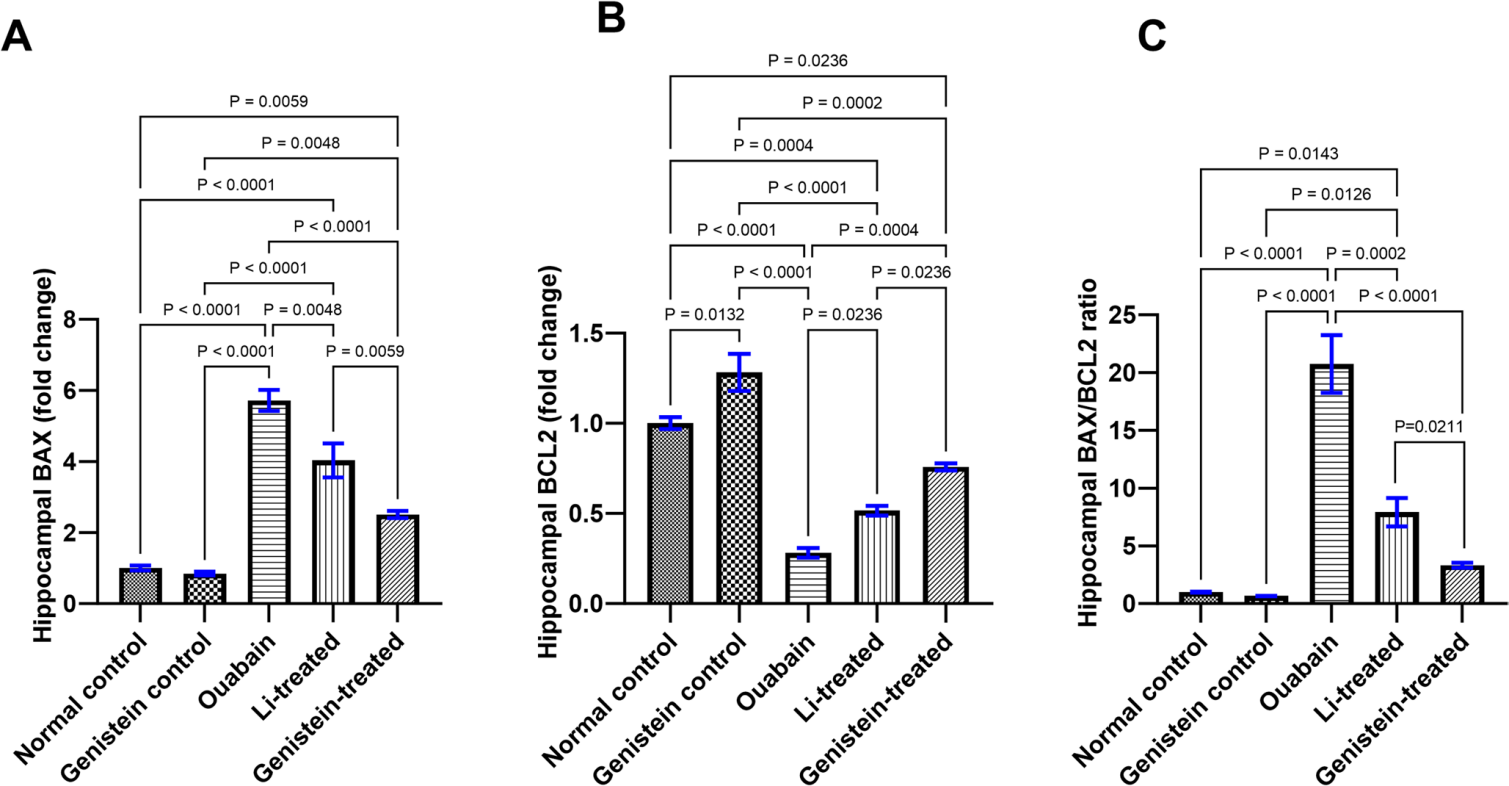
Effect of genistein on hippocampal apoptotic markers in ouabain-induced BD in mice. (A-B) Fold change in expression of BAX and BCL2 respectively. (C) BAX/BCL2 ratio. Statistical differences were examined by ordinary one-way ANOVA test. Each column represents the mean of 3 mice ± SEM. Statistical significance was considered at P <0.05.

Ouabain led to an upregulation of Bax gene expression (*P* < 0.0001, Fig. 8A) and a down-regulation of BCL-2 expression (*P* < 0.0001, Fig. 8B), thereby significantly increasing the BAX/BCL-2 ratio (*P* < 0.0001, Fig. 8C) in the hippocampus. Genistein treatment reduced BAX (*P* < 0.0001, Fig. 8A) and enhanced the expression of BCL-2 (*P* = 0.0004, Fig. 8B), thus significantly decreasing the ratio of BAX/BCL-2 (*P* < 0.0001, Fig. 8C) when compared to the ouabain group. Li treatment restored all of the apoptotic changes brought on by ouabain, evidenced by a significant reduction in BAX (*P* = 0.0048, Fig. 8A) and increased the expression of BCL-2 (*P* = 0.0236, Fig. 8B) in the hippocampus, thereby significantly decreasing the BAX/BCL-2 ratio (*P* = 0.0002, Fig. 8C) when compared to the ouabain group. Genistein's impacts on BAX (*P* = 0.0059, Fig. 8A), BCL-2 (*P* = 0.0236, Fig. 8B) and the BAX/BCL-2 ratio (*P* = 0.0211, Fig. 8C) were superior to those of Li.

### Effect of Genistein on Serotonin Level in Ouabain-Induced BD in Mice

**Fig. 9:**
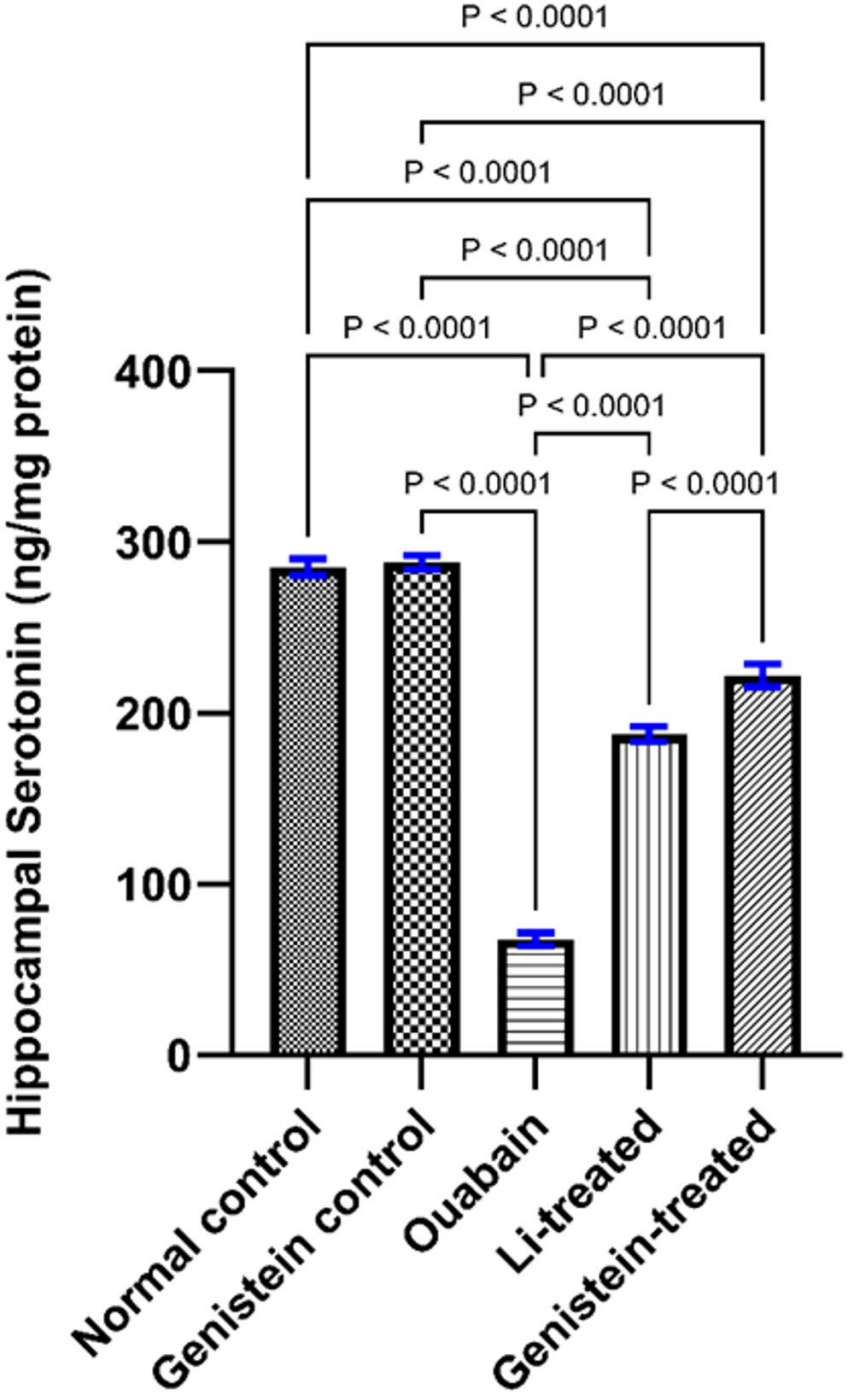
Effect of genistein on hippocampal serotonin level in ouabain-induced BD in mice on the 14th day after ICV injection. Differences in statistics were analyzed by ordinary one-way ANOVA test. Each column represents the mean of 6 mice ± SEM. Statistical variability is considered at *P* < 0.05

Ouabain administration to C57BL/6 mice significantly decreased serotonin levels in the hippocampus (*p* < 0.0001, Fig. 9) when related to the normal control group. Genistein intervention blocked the decrease in serotonin level induced by ouabain (*p* < 0.0001, Fig. 9). Li treatment also prevented the ouabain from lowering serotonin levels in the hippocampus (*p* < 0.0001, Fig. 9) in comparison with the ouabain group. The effects of genistein on serotonin level were superior to those of Li (*p* < 0.0001, Fig. 9).

### Effect of Genistein on Histopathological Alterations in Ouabain-Induced BD in Mice

**Fig. 10:**
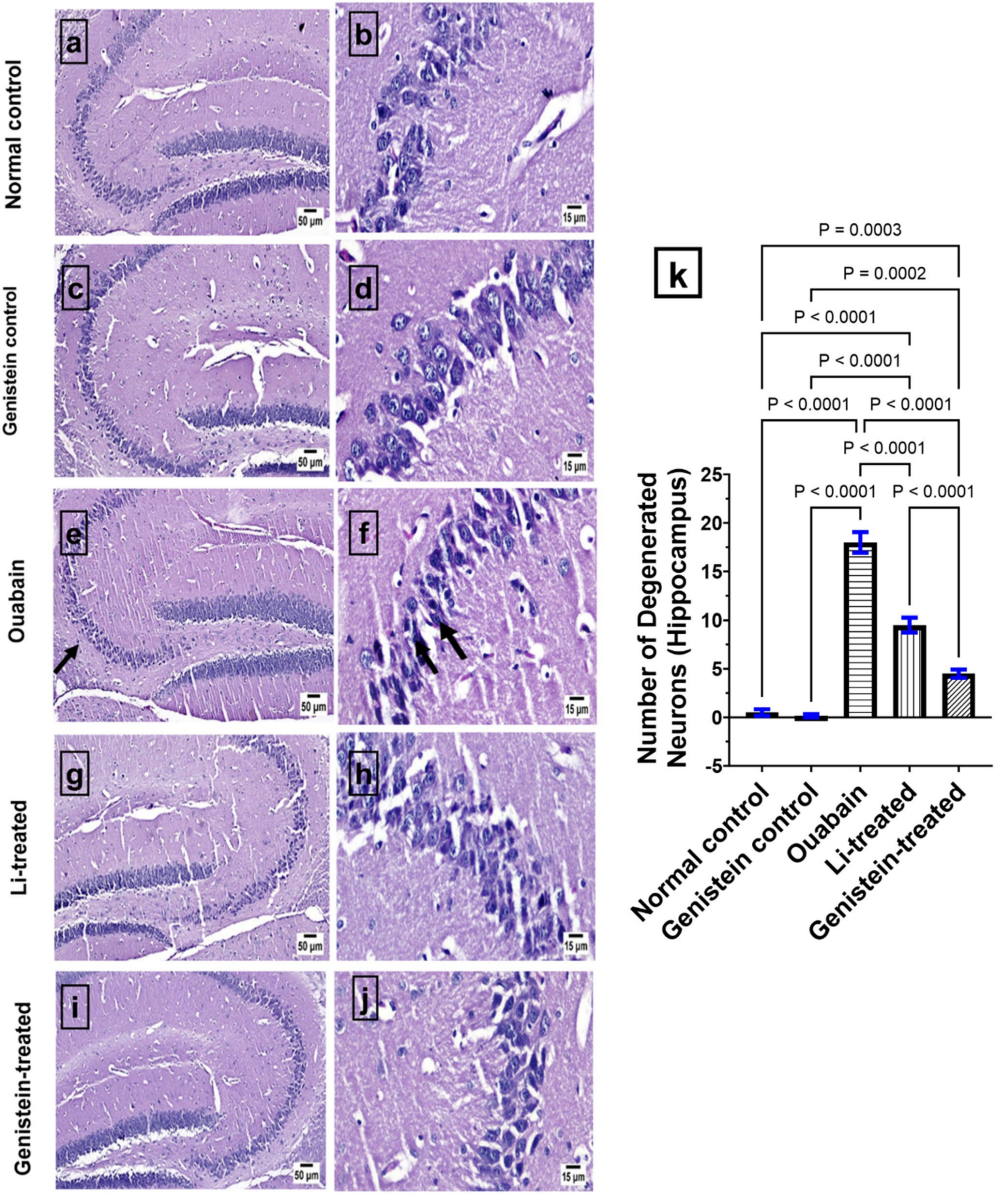
Representative H&E staining photomicrographs of hippocampal sections: (a) and (b) normal control; (c) and (d) Genistein control; (e) and (f) Ouabain group showing dark degenerating neurons (arrows); (g) and (h) Li-treated; (i) and (j) Genistein-treated; (k) Number of degenerated neurons (Hippocampus). Differences in statistics analyzed by ordinary one-way ANOVA test. Each column represents the mean of six non-overlapping fields ± SEM. Statistical variability was considered at *P* < 0.05

**Fig. 11:**
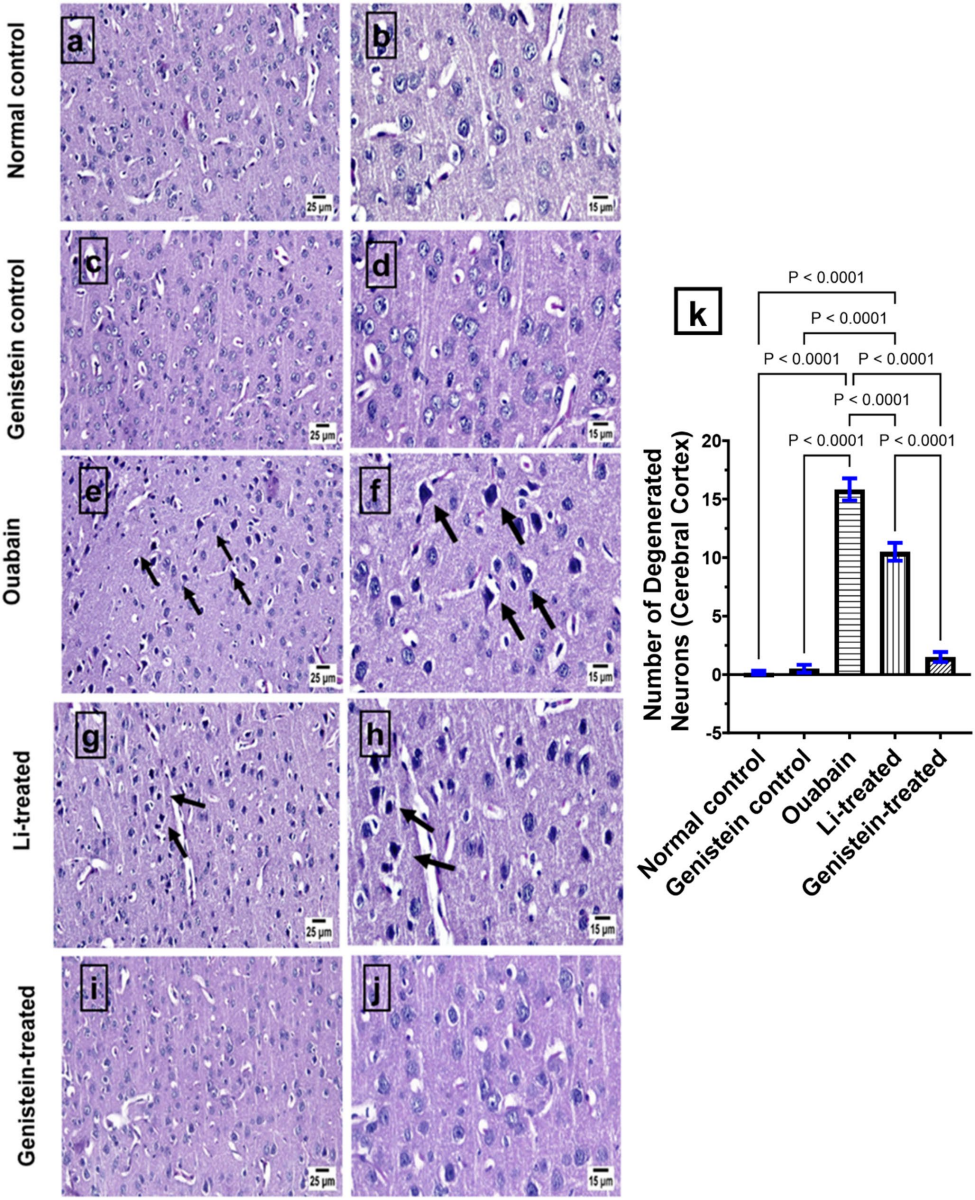
Representative H&E staining photomicrographs of cerebral cortex sections: (a) and (b) normal control; (c) and (d) Genistein control; (e) and (f) Ouabain group showing dark degenerating neurons (arrows); (g) and (h) Li-treated; (i) and (j) Genistein-treated; (k) number of degenerated neurons (cerebral cortex). Differences in statistics analyzed by ordinary one-way ANOVA test. Each column represents the mean of six non-overlapping fields ± SEM. Statistical variability considered at *P* < 0.05

Microscopic examination of brain sections from the control group revealed a normal histological structure of different brain regions, including the hippocampus (Fig. 10) and cerebral cortex (Fig. 11). Similarly, the genistein control group showed an apparently normal hippocampus (Fig. 10) and cerebral cortex (Fig. 11) without any detectable alterations. On the contrary, ouabain group showed dark degenerating neurons within the hippocampus (*p* < 0.0001, Fig. 10) with the presence of numerous degenerating neurons within the cerebral cortex with neuronophagia (*p* < 0.0001, Fig. 11) when related to the normal control group. Marked improvement was detected in the examined brain sections from genistein-treated group in the hippocampus (*p* < 0.0001, Fig. 10) and cerebral cortex (*p* < 0.0001, Fig. 11) when compared to the ouabain group. The Li-treated group showed marked improvement, as the examined sections of hippocampus (*p* < 0.0001, Fig. 10) were apparently normal with the presence of a few dark degenerating neurons within the cerebral cortex (*p* < 0.0001, Fig. 11) in comparison with the ouabain group. The effects of genistein on the hippocampus (*p* < 0.0001, Fig. 10) and cerebral cortex (*p* < 0.0001, Fig. 11) were superior to those of Li.

## Discussion

Genistein is a phytochemical that has been shown to exhibit a multimodal mechanism of action in numerous diseases such as Alzheimer’s disease (AD), Parkinson’s disease (PD), and ischemic stroke [47]. Genistein has been demonstrated to modulate several pathways, some of these pathways affecting Na⁺/K⁺-ATPase signalosome [48].

Ouabain, a reliable model for BD, was employed in this study to mimic BD’s psychological and pathophysiological disturbances and clinical course of BP in humans [49]. Thereby, the purpose of the present research was to assess the neuroprotective actions of genistein in ouabain-induced BD in mice compared to the standard FDA-approved therapy, Li. To evaluate these neuroprotective effects, behavioral, histological, and biochemical experiments were carried out.

In our study 7 days after ICV injection, ouabain increased the motor and exploratory activities in addition to anxious behavior in OFT, which agrees with an earlier report demonstrating that ouabain increased motor, exploratory activity and anxiety behaviors [50]. Such results are consistent with the fact that inhibiting Na⁺/K⁺-ATPase enzyme increases the membrane potential nearer to the threshold, rising neuron excitability and inducing BD manic episodes [10].

Meanwhile, genistein treatment in this study demonstrated a reduction in manic and anxious behavior 7 days after ICV injection of ouabain. This is consistent with earlier studies where genistein exerted anxiolytic-like effect in ovariectomized rats with long-term lack of ovarian hormones [51]. Herein, Li reversed the manic behavior observed in ouabain -treated mice, which is compatible with a past study demonstrating the anti-manic effects of Li in the ouabain model [52].

However, by 14 days post-ouabain injection, motor and exploratory functions in the OFT were notably decreased, which concurs with an earlier study [53]. Additionally, depressive-like behavior emerged, consistent with a previous report [54]. Decreased motor activity and anhedonia reported herein after 14 days of ouabain injection can be attributed to the prolonged effect of ouabain on neuronal excitability that might subsequently decrease the control of the resting potential, interfering in neuronal depolarization, decreasing neuronal signaling velocity, and ultimately, declining synaptic efficiency and triggering a depressive episode [55].

Conversely, genistein treatment in the current study significantly improved motor activity in the OFT 14 days after ouabain injection. Our findings are in agreement with an earlier study where genistein enhanced motor functions in a mouse model of PD [56] and in a transgenic mouse paradigm of amyotrophic lateral sclerosis (ALS) [57]. This can be attributed to the fact that soy flavones, especially genistein, have been previously demonstrated to exert anti-fatigue and anti-hypoxic properties in experimental animals [58]. Moreover, genistein has been previously reported to protect experimental animals against exhaustive exercise [59]. 

Furthermore, genistein demonstrated in this report a substantial improvement in depressed behavior, as evidenced by a decrease in anhedonia and behavioral despair. These findings align with previous research where genistein caused a considerable increase in the sucrose preference ratio in rats subjected to chronic mild stress [60] and reduced immobility time in FST in ovariectomized female rats [61]. These anti-depressive effects of genistein can be explained in light of the fact that genistein has been demonstrated to modulate serotonergic metabolism in the hippocampus of ovariectomized rats [62]. Likewise, Li-treated group showed a significant reduction in depressive behavior, which is consistent with previous preclinical research demonstrating antidepressant characteristics for Li in BD [63]. Moreover, Li has been previously demonstrated to reduce anhedonia in rats exposed to repeated unavoidable stress [64] and ameliorate behavioral despair induced by acute neurogenic stress in mice [65]

The neurotransmitter serotonin regulates many brain functions, including stress, mood and movement [66]. Reduced serotonin levels have been utilized as a potential hallmark for the early detection of BD in individuals [67]. In the current study, ICV injection of ouabain considerably reduced hippocampal levels of serotonin. A previous report has demonstrated a similar effect on serotonin levels with ouabain administration [68]. Moreover, Na⁺/K⁺-ATPase inhibition has been reported to inhibit kynurenine pathway resulting in a subsequent decrease in tryptophan, which is the synthetic precursor of serotonin [69]. Such an effect can explicate the serotonin depletion and depressive-like behavior observed herein with ouabain administration.

Herein, genistein treatment significantly increased serotonin levels in the hippocampus, which was consistent with the previously reported antidepressant effect of genistein in ovariectomized rats [70] and a post-traumatic stress disorder rat model [71]. Such an increase in serotonin concentrations in response to genistein treatment could be partly explained by its capacity to decrease serotonin turnover and regulate monoamine metabolism, especially on a serotonergic level [72]. The current report also demonstrates that Li treatment increased hippocampal serotonin levels in ouabain-induced BD, which is in agreement with a previous study [73].

The current work, ouabain, has also been shown to adversely affect body weight. Weight loss has been previously reported in a previous study in ouabain-induced BD [68]. Such a reduction in body weight can be explained by the increased motor activity observed in the manic phase (day 1 to day 7) followed by the reduced food intake and anhedonia observed in the depressive phase (day 8 to day 14).

On the other hand, mice treated orally with genistein exhibited a remarkable restoration of body weight, aligning with previous findings where genistein improved body weight, serum glucose, and triglyceride levels in hyperphagic, obese male and female mice [74]. Moreover, genistein has been reported to collectively maintain an efficient metabolic homeostatic status, which improves the overall health and growth in mice with diet-induced prediabetes [75]. Similarly, in the present report, Li treatment also resulted in a substantial rise in weight gain, consistent with earlier research in a mild stress-exposed rat‏ model [76].

Herein, aberrations induced by ouabain administration were investigated both on the level of behavioral manifestations and intracellular signaling cascades. Ouabain is believed to not only block the Na⁺/K⁺-ATPase by attaching to the enzyme’s extracellular domains [77] but also forms various signalosomes which trigger a diverse range of cellular signaling pathways that modulate neuronal plasticity and survival. In fact, Na⁺/K⁺-ATPase signalosomes are crucial for neuronal functioning via preserving and restoring the electrochemical gradient after every depolarization in order for neurons to return to their resting state [12].

Through the binding of the ouabain to Na⁺/K⁺-ATPase, a series of protein–protein interactions occur, prompting various signaling pathways triggering actions in Na⁺/K⁺-ATPase beyond its pumping function [12]. Ouabain induces Na⁺/K⁺-ATPase-mediated stimulation of Src and induces the transactivation of EGFR[ 77] along with phosphorylating and activating the ERK1/2 signaling pathway [78], which in turn can activate NF-κB. This is consistent with a previous study in which ouabain activated NF-κB in cultured cardiac myocytes[79]. NF-κB acts as a target for reactive oxygen species (ROS) and an inducer of pro-inflammatory cytokines IL-1β[80] and iNOS expression, which makes NF-κB a component of the development of neuroinflammation in the oxidative stress process in BD [81].

In our study, ouabain injected group exhibited a substantial increase in the phosphorylated forms of Src, EGFR, and ERK1/2 protein. Such results can be attributed to the pleiotropic effects exerted by ouabain, where ouabain changes the interaction of Na⁺/K⁺-ATPase with neighboring membrane proteins, resulting in activation of Src, then transactivation of EGFR, and finally increased phosphorylation levels of ERK1/2 [12].

The present report has demonstrated that genistein administration resulted in a noticeable decline in the phosphorylation of Src, EGFR, and ERK1/2. This was in line with a previous study in which genistein decreased EGFR activation in HCT 116 colon cancer cells [82]. Additionally, a prior investigation revealed that the phosphorylation levels of Src, EGFR, and ERK1/2 were downregulated by genistein in SGC-7901 cells [83]. In addition, Li treatment led to a decrease in the amounts of phosphorylated Src, EGFR, and ERK1/2, which agrees with several previous studies in titanium nanoparticle-stimulated inflammatory model in rats [84], as well as in human breast epithelial cells [85].

In this study, ouabain caused a marked decline in phospho-CREB, followed by a substantial decrease in BDNF levels. This was consistent with previous reports demonstrating decreased BDNF levels in BD patients [86]. In addition, recent studies have found that the expression of the BDNF [50,53 ] and CREB phosphorylation were downregulated in the hippocampus and frontal cortex of the animal models of ouabain-induced BD. However, genistein treatment significantly increased phospho-CREB and BDNF levels 14 days after ouabain injection. Our findings are in agreement with previous studies showing the beneficial effect of genistein treatment, which ameliorated memory impairment in the mice by significantly increasing BDNF expression level and CREB phosphorylation in lipopolysaccharide (LPS)-treated mice [87]. Another study has showed that, treatment with genistein significantly increased BDNF level in rats with chronic mild stress, which confirms the neuro-regenerative role of genistein [60]. Herein, treatment with Li increased the hippocampal levels of BDNF and phospho-CREB which is in line with a previous report on ouabain-induced BD in rats [50].

Oxidative stress has been proven to contribute to diminished neuroplasticity, neurogenesis, and increased apoptosis and neurodegeneration in BD [88]. In this research, ouabain administration increased the hippocampal MDA levels and decreased SOD and GSH levels. SOD and GSH are the main defenses involved in cellular protection against damage caused by oxygen-derived free radicals [89]. Previous studies suggested that the ouabain-induced production of superoxide radicals and damage to the mitochondrial membrane affect the entire cell ref. This may help clarify some of the oxidative damage that the proteins and lipids have sustained, which was proved by the elevated MDA levels observed in experimental animal models [90].

Conversely, a noticeable rise in SOD and GSH levels and a lowering in MDA concentrations were observed after genistein treatment. These findings are in agreement with a prior report that found that giving amnesic mice genistein has successfully reduced stress caused by oxidation in their hippocampal regions [91]. Another study emphasized the possible neuroprotective advantages of genistein against Aβ-induced oxidative stress-mediated in rat primary hippocampal neurons, where genistein decreased the accumulation of ROS and decreased the contents of MDA in the rats^,^ hippocampus [92]. Altogether, genistein's antioxidant effects is thought to be based on its capacity to act as a ROS scavenger and as a stimulus for the antioxidant defense systems such as SOD and GSH [93]. Moreover, Li increased both SOD and GSH levels and decreased MDA levels. These results coincide with previous studies showing the antioxidant like properties of Li [9].

Another hallmark of BD is the presence of pro-inflammatory milieu, leading to an increased production of peripheral inflammatory cytokines which was previously reported, and it has been proposed to contribute to the pathophysiology of BD [94]. Herein, ouabain administration has caused a significant increase in IL-1β, NF-κB as well as iNOS levels. It is well known that ouabain blocks the Na⁺/K⁺-ATPase activity, leading to increased intracellular Na⁺ concentration that transactivates Na⁺/Ca²⁺ exchangers. This process leads to elevated cytosolic Ca²⁺ levels, generating Ca²⁺ waves that are enough to provoke subsequent signaling events, including the activation of NF-κB [95]. An earlier study has confirmed that ICV administration of ouabain caused stimulation of NF-κB and a subsequent elevation in the inflammatory cytokines and iNOS level in the rat hippocampus [96]. Additionally, another study has shown ouabain as a potent inducer of nitric oxide formation, iNOS upregulation, and increased production of ROS in cultured adult rat ventricular myocytes [97].

Oral administration of genistein in the current study notably reduced NF-κB, IL-1β and iNOS levels. Genistein has been shown to inhibit the activation of NF-κB signaling pathway, which is recognized for preserving homeostatic equilibrium between cell survival and apoptosis [98]. Our results are in line with a previous study where genistein treatment significantly suppressed NF-κB and iNOS and inhibited pro-inflammatory cytokines in the cortex and hippocampus of mice subjected to chronic sleep deprivation (CSD) [99]. This was also consistent with another study where genistein treatment alleviated oxidative stress and inflammation via the suppression of NF-κB pathway in neonatal mice [100]. In the current report, Li treatment reduced NF-κB, IL-1β and iNOS levels elicited by ouabain. Such results are consistent with former research conducted in LPS-induced inflammation in rats [101].

There is increasing evidence suggesting that dysregulation of neurotrophic signaling cascades observed in BD is associated with apoptosis and contributes to brain atrophy in BD patients [102]. Herein, ouabain-treated group showed a decreased BCL-2 expression with a concurrent rise in the expression of the BAX apoptotic marker. This is consistent with earlier studies where ouabain upregulated the expression of BAX and decreased the expression of BCL-2 in rats [68] and in human osteosarcoma cells [103].

In the present work, genistein treatment elicited a neuroprotective effect by increasing the expression of BCL-2 and decreasing BAX. In a previous report, genistein has managed to attenuate neuronal apoptosis in ovariectomized rats [104]. Furthermore, genistein greatly enhanced lifespan of cells, lowered the quantity of cells that undergo apoptosis, and upregulated BCL-2 in neurons of the hippocampus after β-induced oxidative stress insults [92]. Furthermore, Li treatment decreased the BAX/BCL-2 ratio which coincides with a previous report [105].

The histological analysis showed that ouabain administration causes neuronal death in the hippocampus. This is congruent with prior research, which found that ouabain intoxication increased the number of neuroglial cells and caused changes in the neuronal morphology [68]. On the other hand, genistein treatment reversed these changes showing marked improvement in the examined hippocampal brain section. This aligns with several prior studies where genistein demonstrated neuroprotective effects in ischemic brain injury [106], in a focal cerebral ischemia rat model [107], and in rats with streptozotocin-induced diabetes mellitus [108]. Li treatment reversed neuronal death in ouabain-treated mice, which is in agreement with a previous report on LPS-induced neurotoxicity in rats [109].

Throughout the whole experiment, the effects of both genistein and Li were comparable and no statistically significant variations exist among them. However, the effects of genistein were superior to those of Li in the distance travelled in FST, and in mobility time on days 7 and 14 in OFT. Moreover, the effects of genistein on NF-κB, IL-1β, iNOS, BAX, BCL-2, the BAX/BCL-2 ratio, MDA, BDNF and serotonin level were more evident than that caused by Li . However, the effects of Li were superior to genistein in the case of phospho-EGFR and phospho-Src.

In the present study female mice were not included, which limits the understanding of our observations in both sexes, resulting in limited translational comparisons, especially since BD affects both sexes. Therefore, we urge future studies to incorporate gender as a confounding factor in their experimental design.

## Conclusion

The provided biochemical, neurobehavioral, and histopathological outcomes exhibited for the first time an intricate neuroprotective mechanism for genistein in ouabain-induced BD paradigm. The orally administrated genistein, a simple trihydroxy isoflavone, protected against ouabain-induced neurotoxicity by activating the Na⁺/K⁺-ATPase signalosome, promoting serotonin neurotransmission and ameliorating the oxidative stress milieu, neuro-inflammation, and apoptosis. Similar effects were perceived with Li, a popular human mood adjuster used for the management of BD . Collectively, such results present genistein as a promising candidate for the treatment of BD.

## Supplementary Information

Below is the link to the electronic supplementary material.

Supplementary Material 1

## Data Availability

The data supporting this article have been included as part of the Supplementary Information.

## Abbreviations

5-HT: 5-Hydroxytryptamine or serotonin
aCSF: Artificial cerebrospinal fluid
AD: Alzheimer’s disease
ALS: Amyotrophic lateral sclerosis
ANOVA: One-way analysis of variance
BAX: BCL-2-associated X protein
BBB: Blood brain barrier
BCL2: B-cell lymphoma/leukemia 2
BD: Bipolar disorder
BDNF: Brain derived neurotrophic factor
CMC-Na: Sodium salt of carboxy methylcellulose
CRE: cAMP response element
CREB: cAMP response element-binding protein
ELISA: Enzyme linked immunosorbent assay
FC: Fold change
FST: Forced swim test
GABA: Gamma aminobutyric acid
GAPDH: Glyceraldehyde 3-phosphate dehydrogenase
GSH: Glutathione
H & E: Hematoxylin and eosin
ICV: Intracerebroventricular
IgG: Immunoglobulin G
IL-1β: Interleukein-1 beta
iNOS: Inducible nitric oxide synthase
I.P: Intraperitoneal injection
Li: Lithium
LPS: Lipopolysaccharide
MDA: Malondialdehyde
NF-κB: Nuclear factor kappa B
OD: Optical density
OFT: Open field test
PBS: Phosphate-buffered saline
PD: Parkinson’s disease
p-CREB: Phosphorylated cAMP response element-binding protein
p-EGFR: Phosphorylated epidermal growth factor receptor
p-ERK: Phosphorylated extracellular signal-regulated kinase
P.O.: Per os, or by mouth
p-Src: Phosphorylated proto-oncogene tyrosine-protein kinase
qRT-PCR: Quantitative reverse transcriptase polymerase chain reaction
ROS: Reactive oxygen species
SDS-PAGE: Sodium dodecyl sulfate–polyacrylamide gel electrophoresis
SEM: Standard error of the mean
SOD: Superoxide dismutase
SPT: Sucrose preference test
