# Supplementary material for: Genistein Exerts Neuroprotective Effects in an Ouabain-Induced Model of Bipolar Disorder: Behavioral and Molecular Insights
Source: Neurochem Res. 2025 Dec 8;51(1):10. doi: 10.1007/s11064-025-04597-3 (PMC12682918; doi:10.1007/s11064-025-04597-3)
Supplement: Supplementary file 1 — Supplementary Material 1 [file 11064_2025_4597_MOESM1_ESM.docx]

**Supplementary material (Raw data of Western blot)**


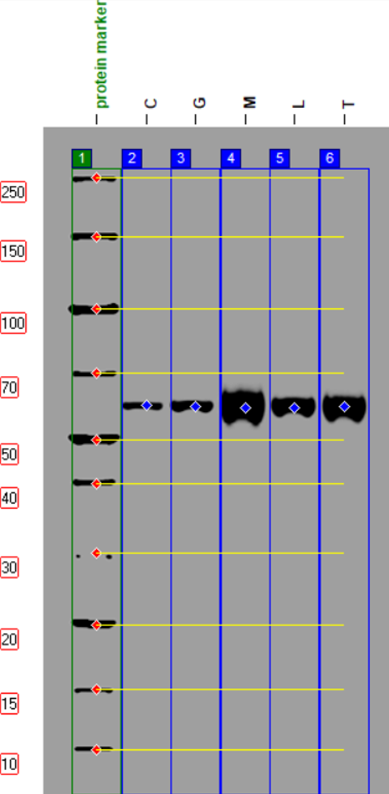

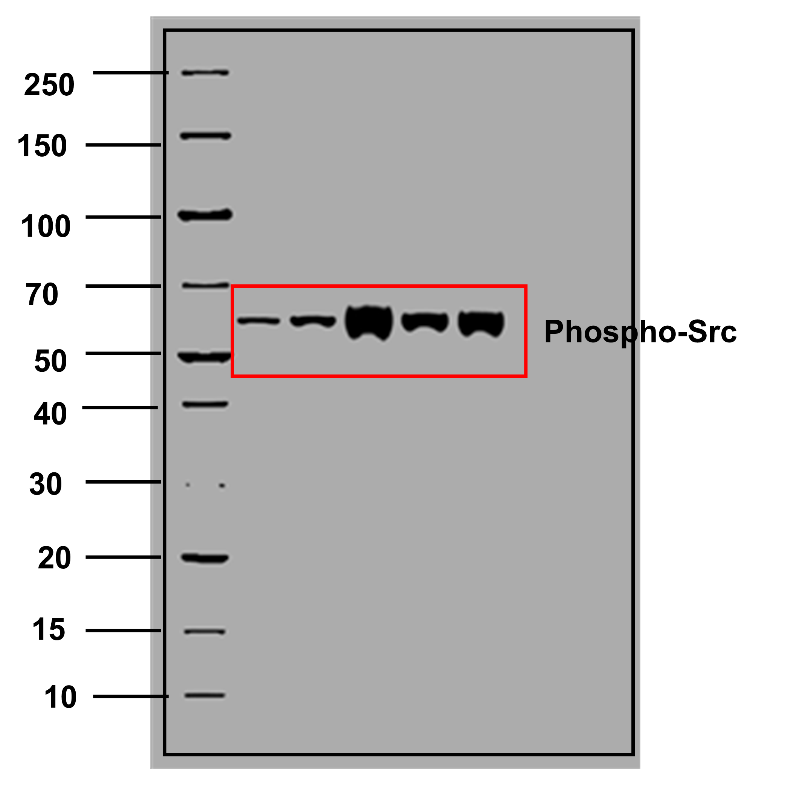


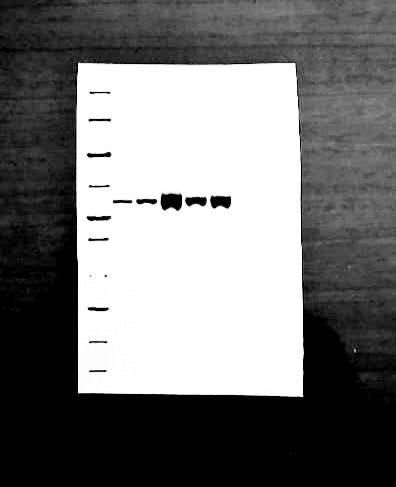


Figure 1: Anti-Src Phosphorylated form expression (1).


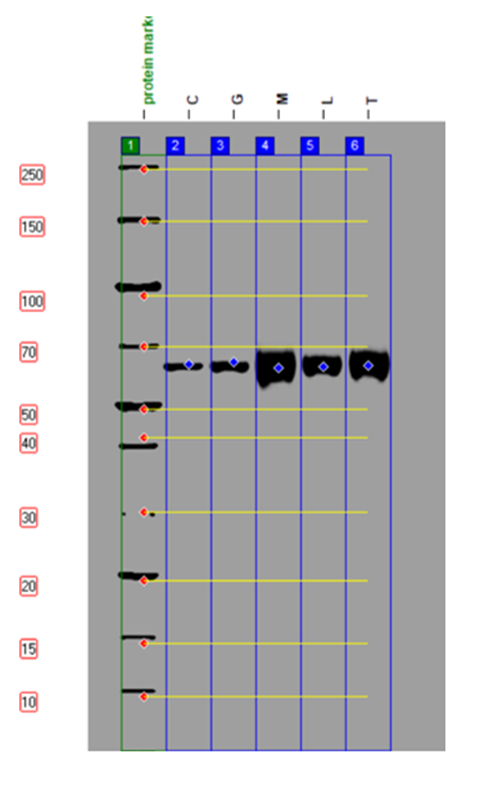

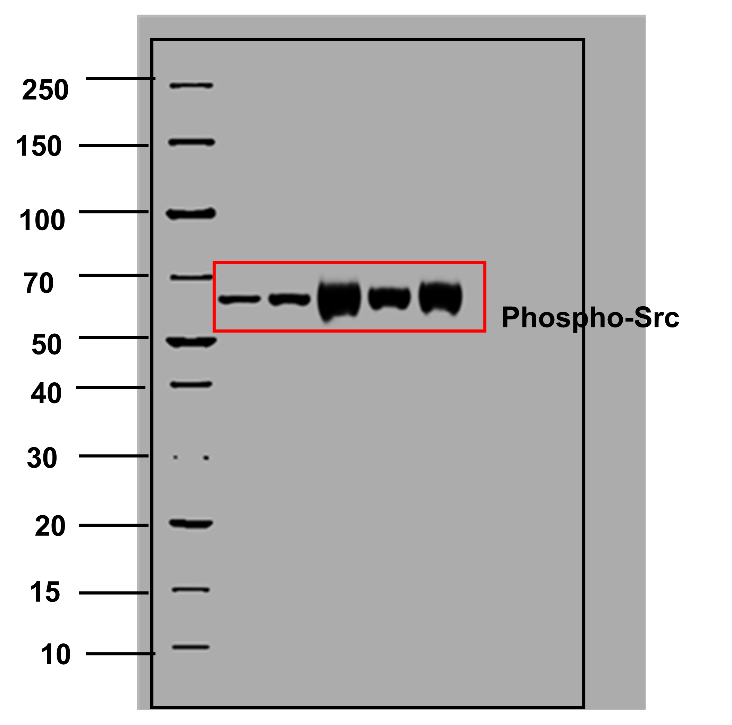

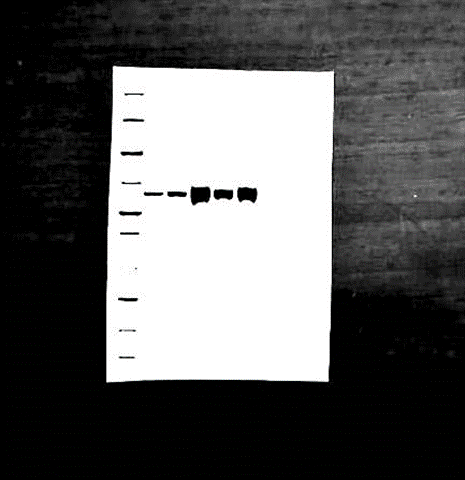


Figure 2: Anti-Src Phosphorylated form expression (2).


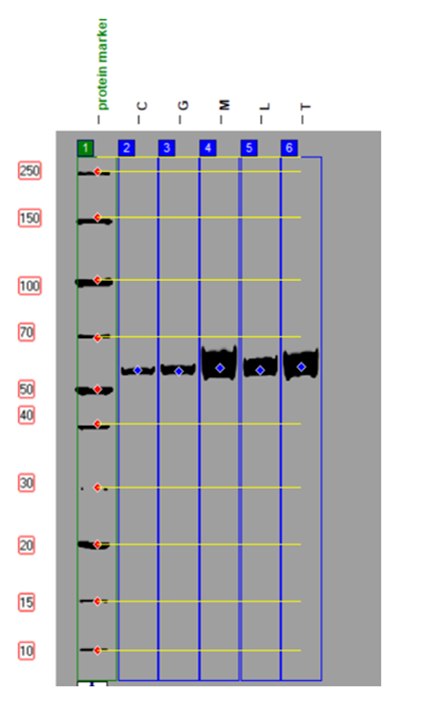

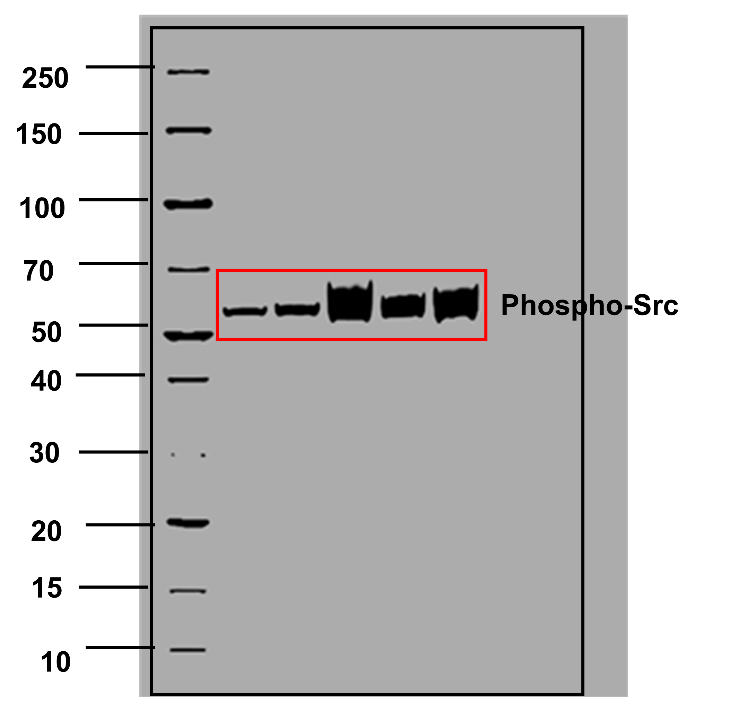

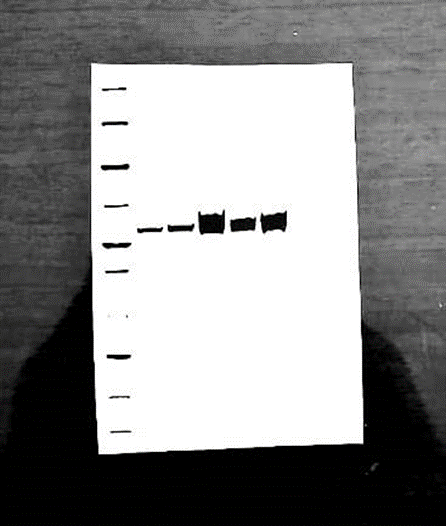


Figure 3: Anti-Src Phosphorylated form expression (3).


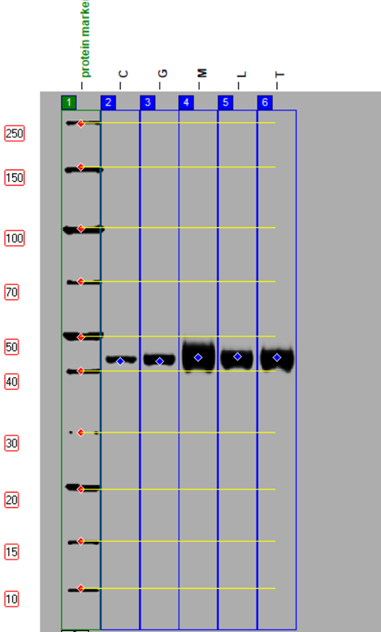

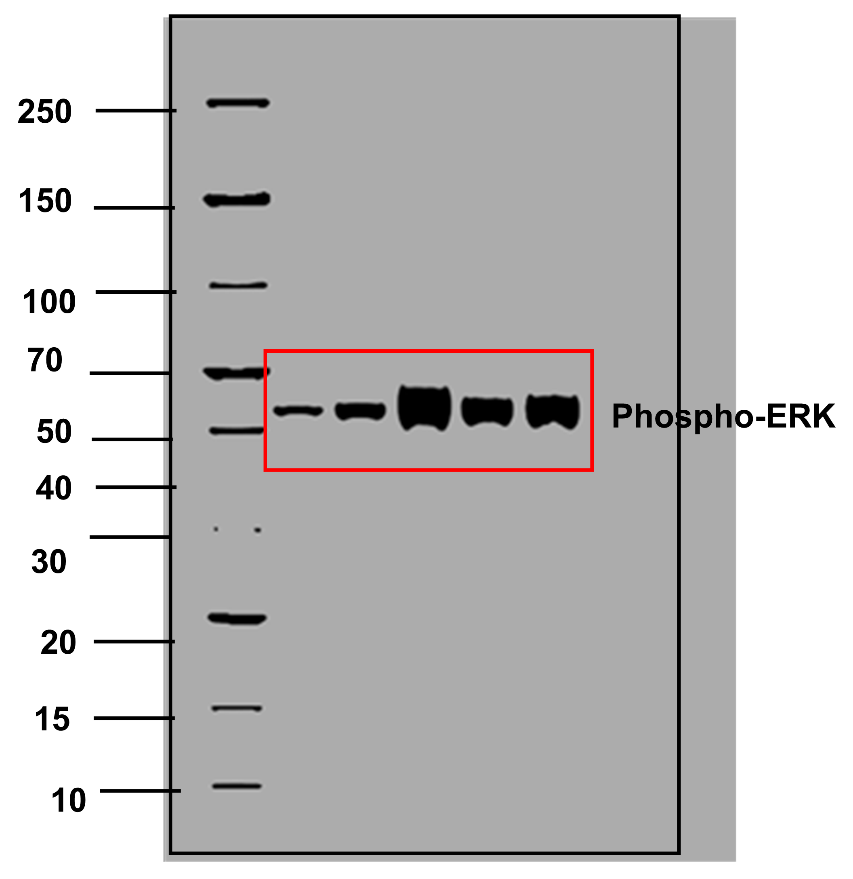

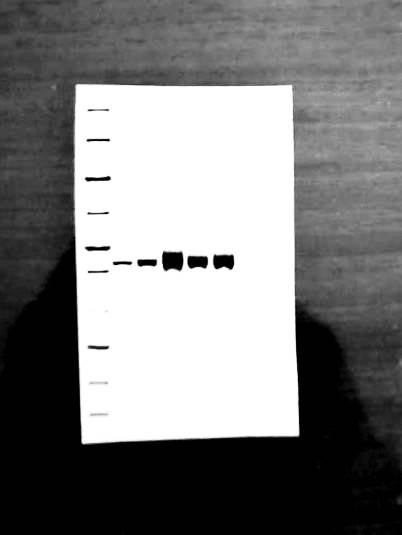


Figure 4: Anti-ERK Phosphorylated form expression (1).


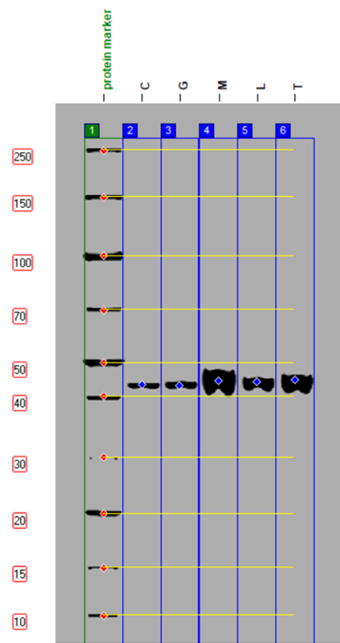

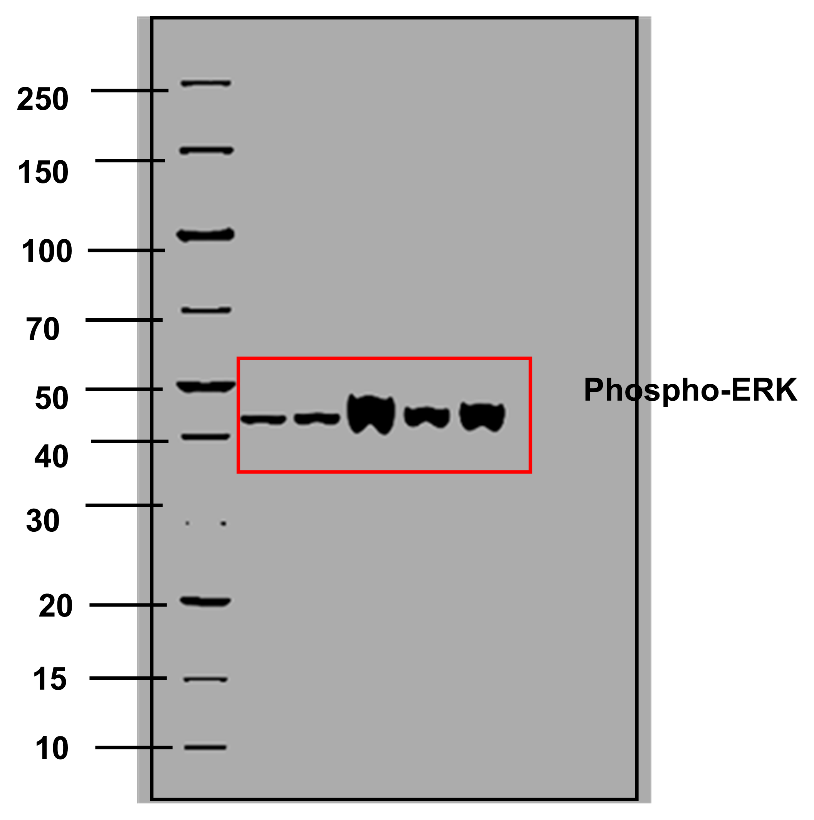

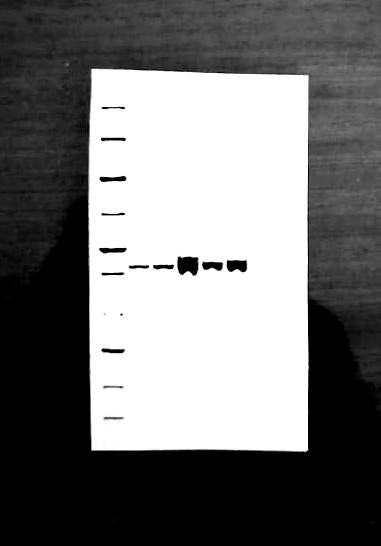


Figure 5: Anti-ERK Phosphorylated form expression (2).


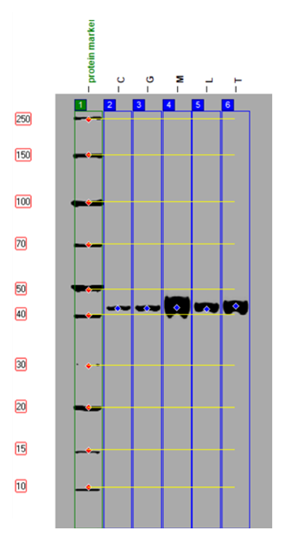

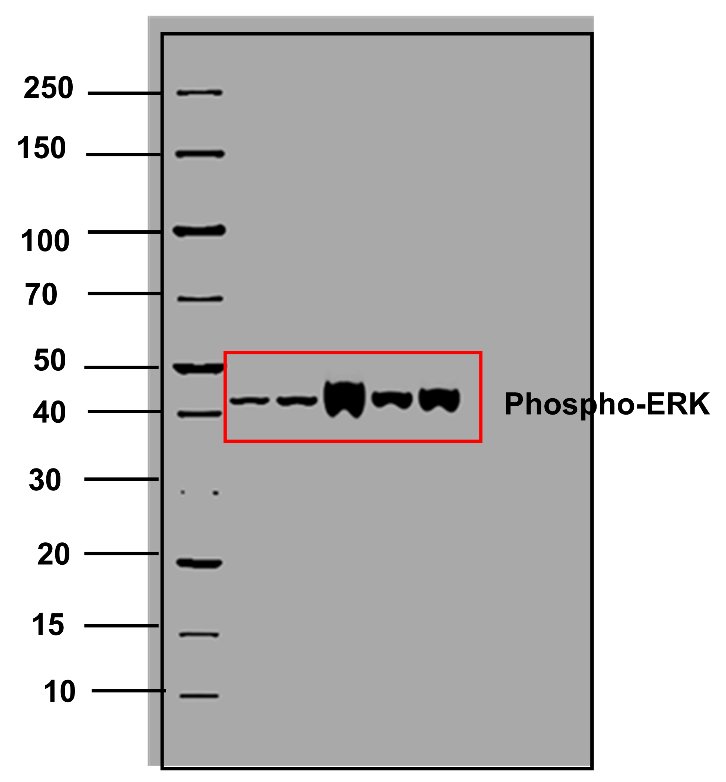

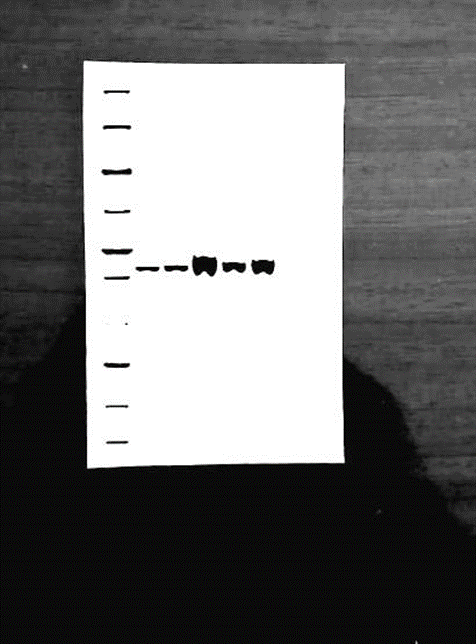


Figure 6: Anti-ERK Phosphorylated form expression (3).


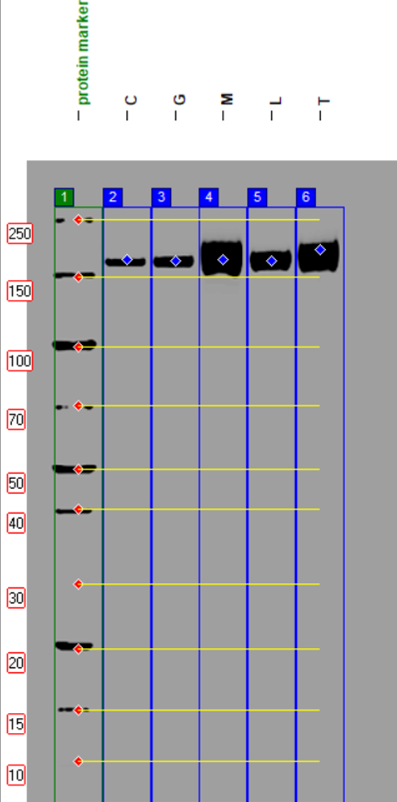

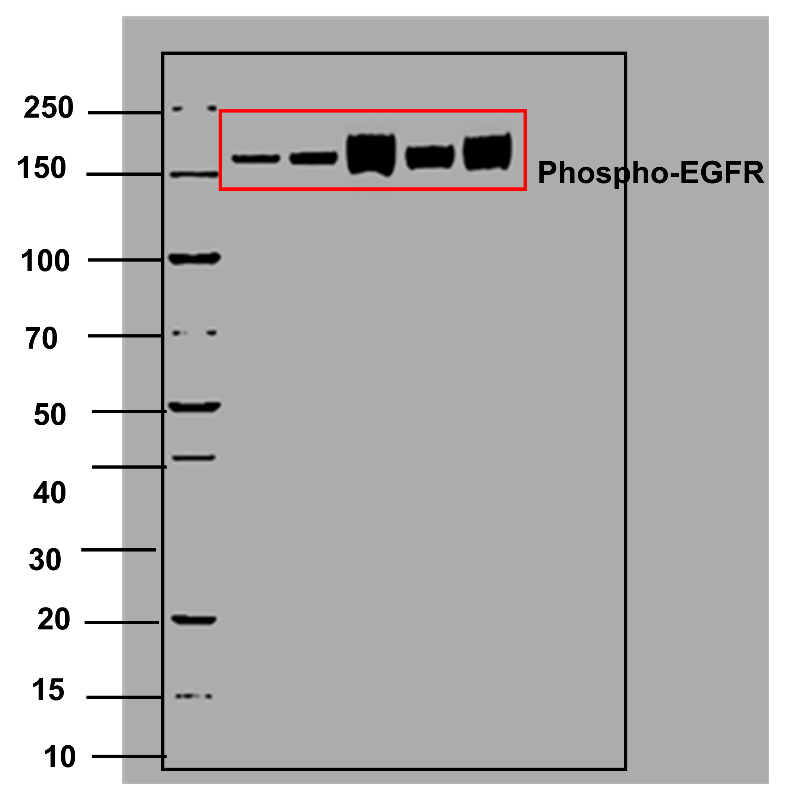

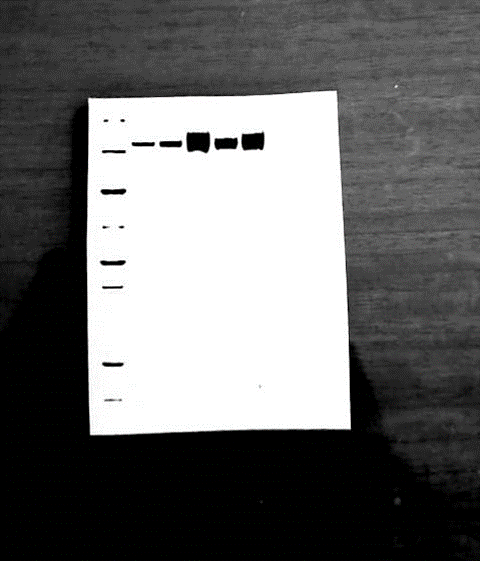


Figure 7: Anti- EGFR Phosphorylated form expression (1).


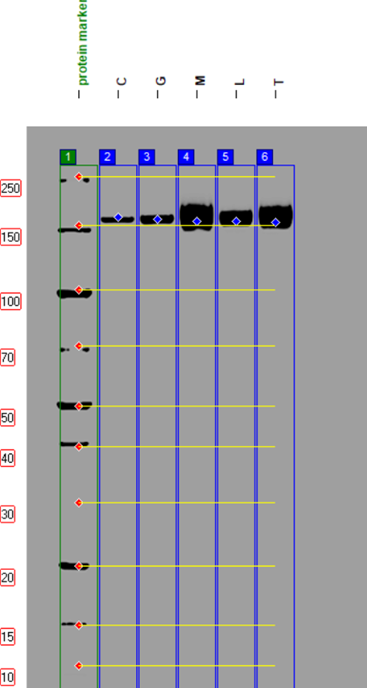

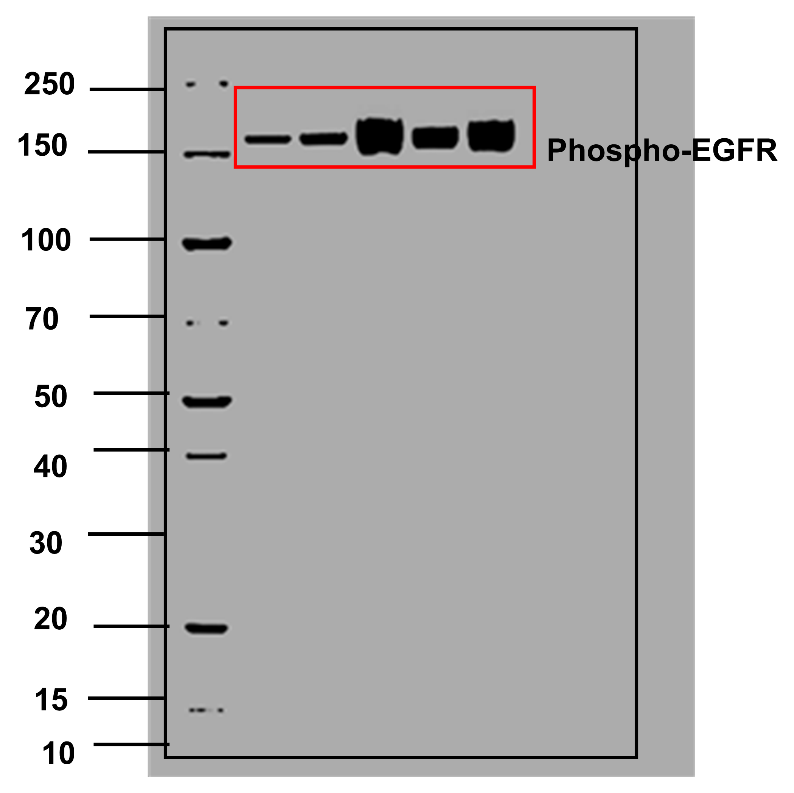

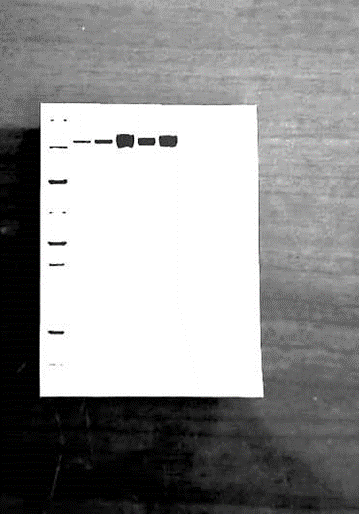


Figure 8: Anti- EGFR Phosphorylated form expression (2).


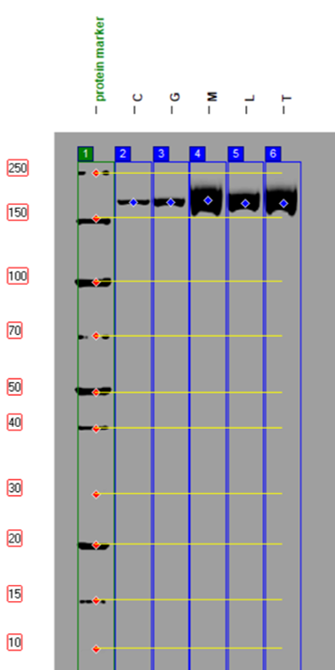

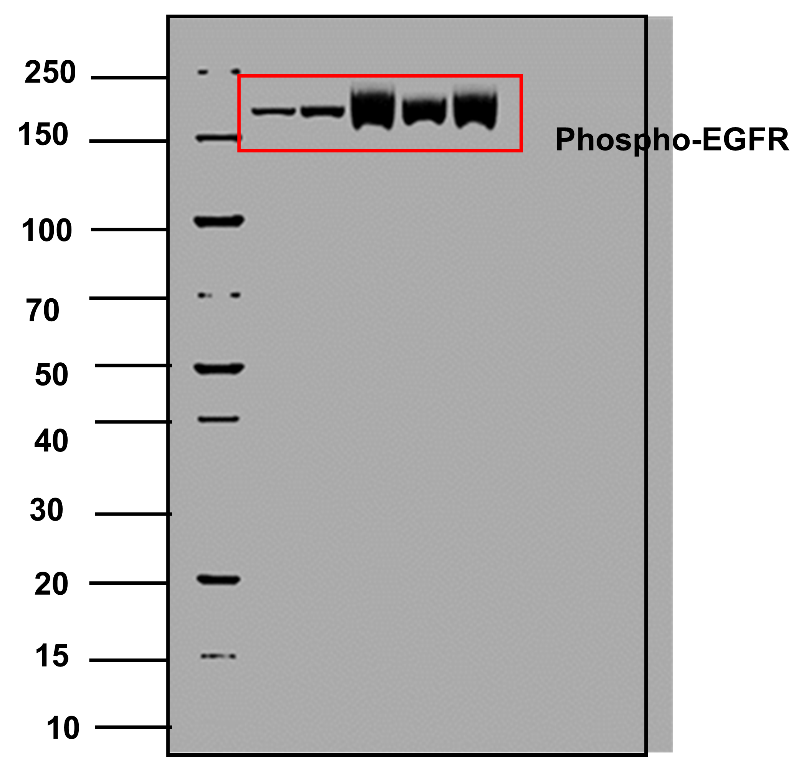

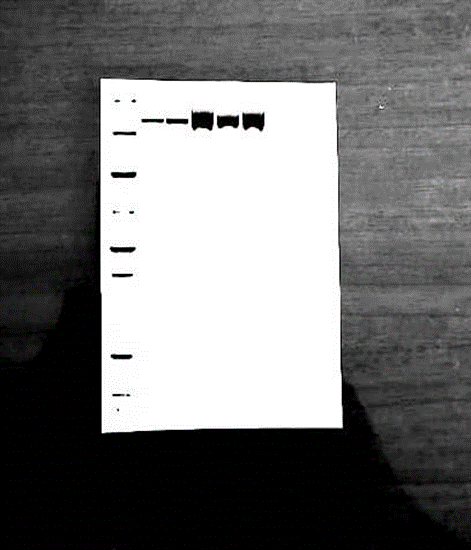


Figure 9: Anti- EGFR Phosphorylated form expression (3).


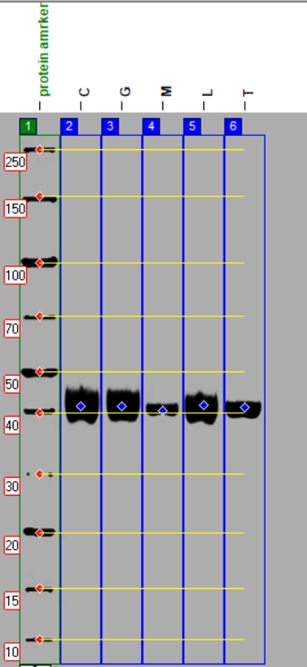

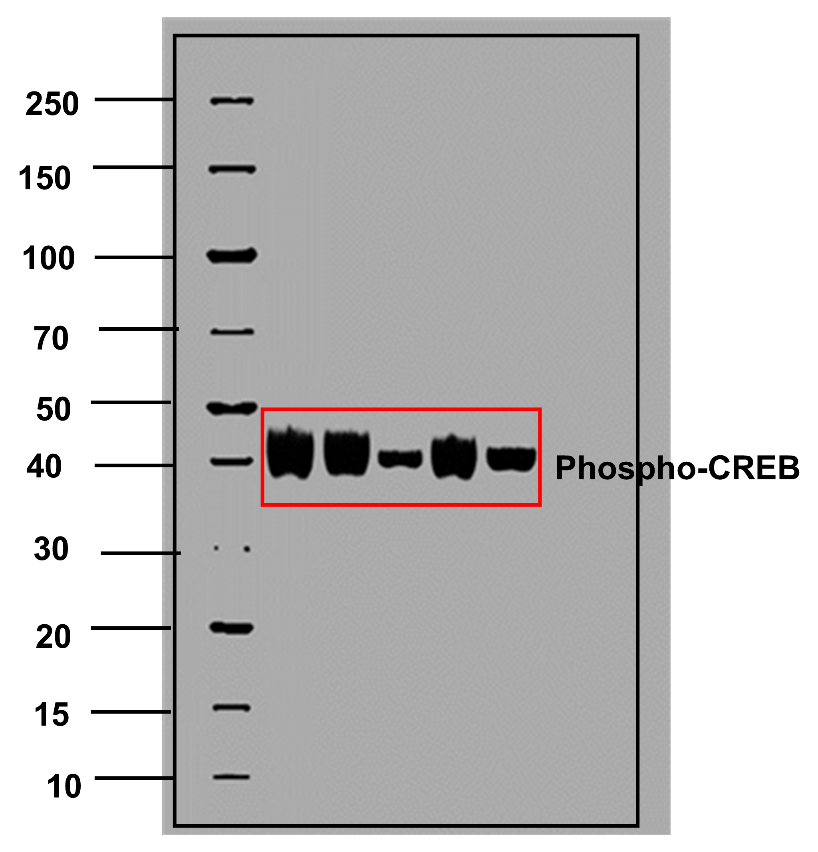

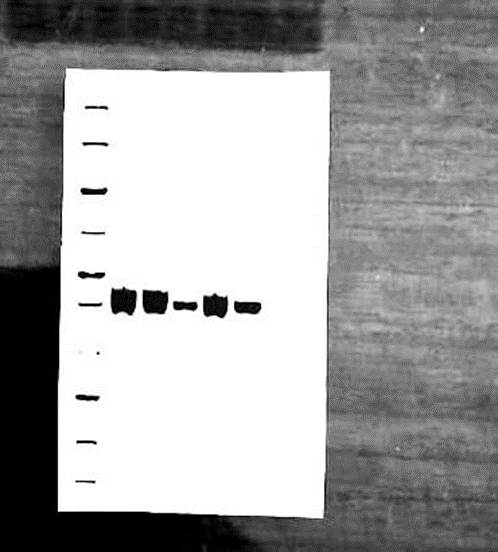


Figure 10: Anti-CREB Phosphorylated form expression (1).


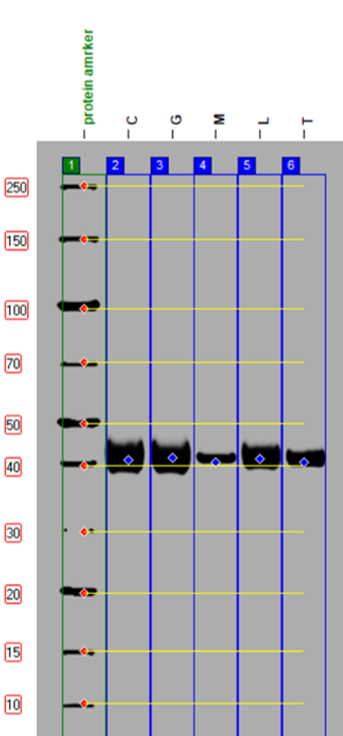

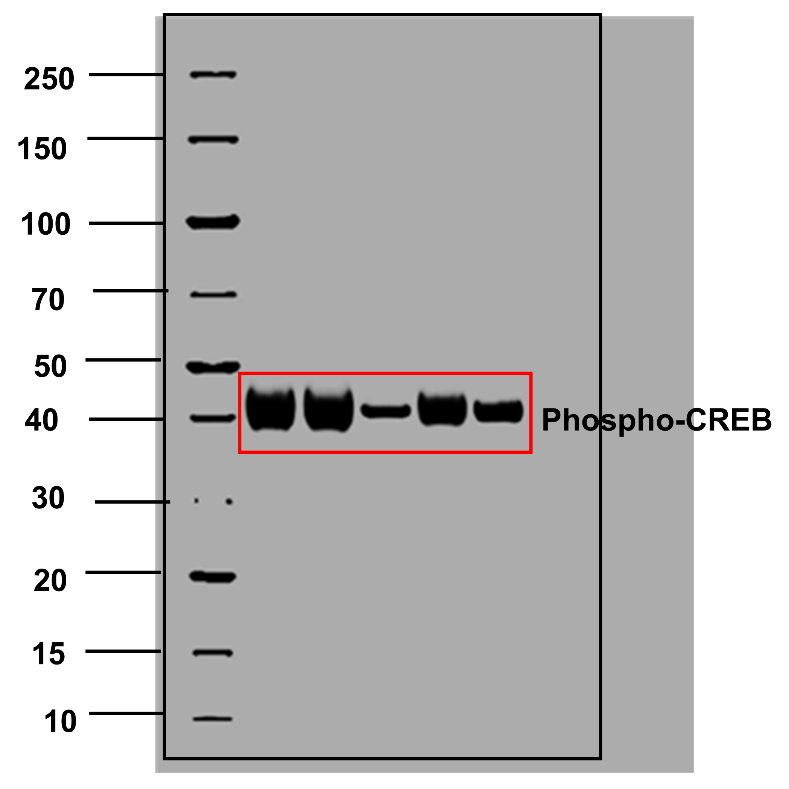

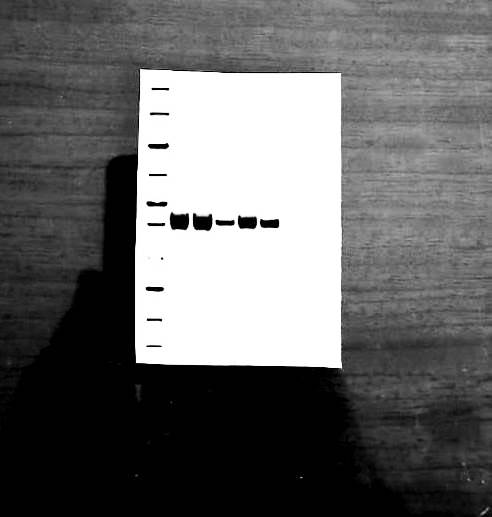


Figure 11: Anti-CREB Phosphorylated form expression (2).


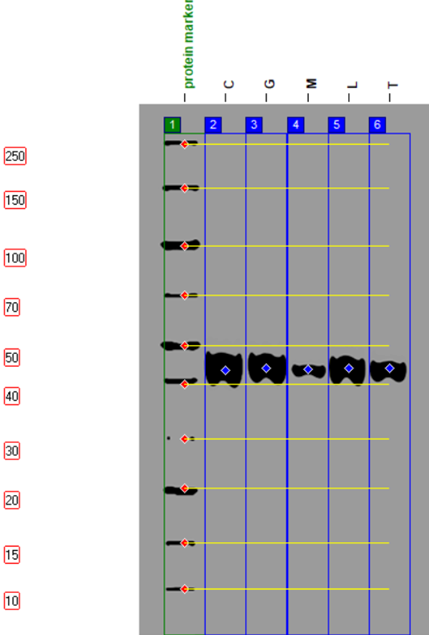

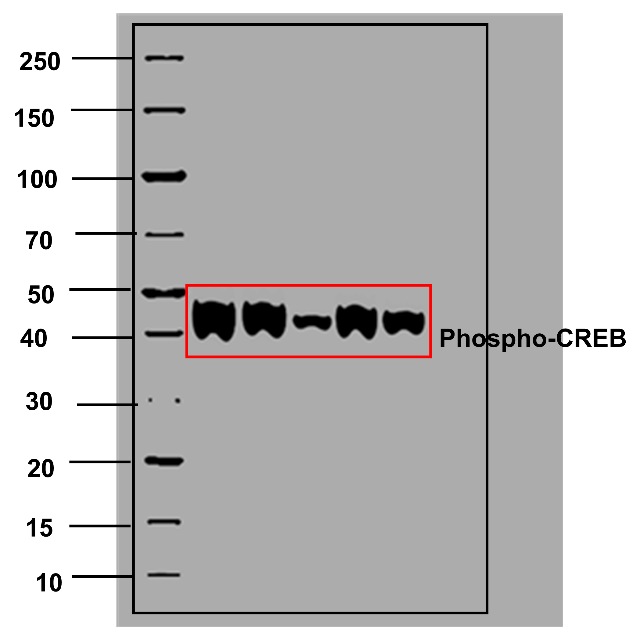

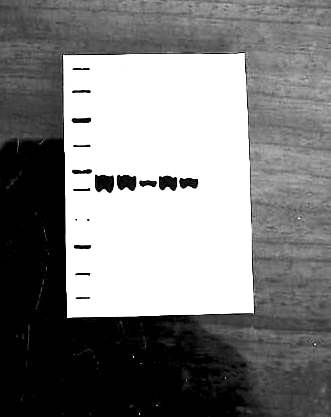


Figure 12: Anti-CREB Phosphorylated form expression (3).


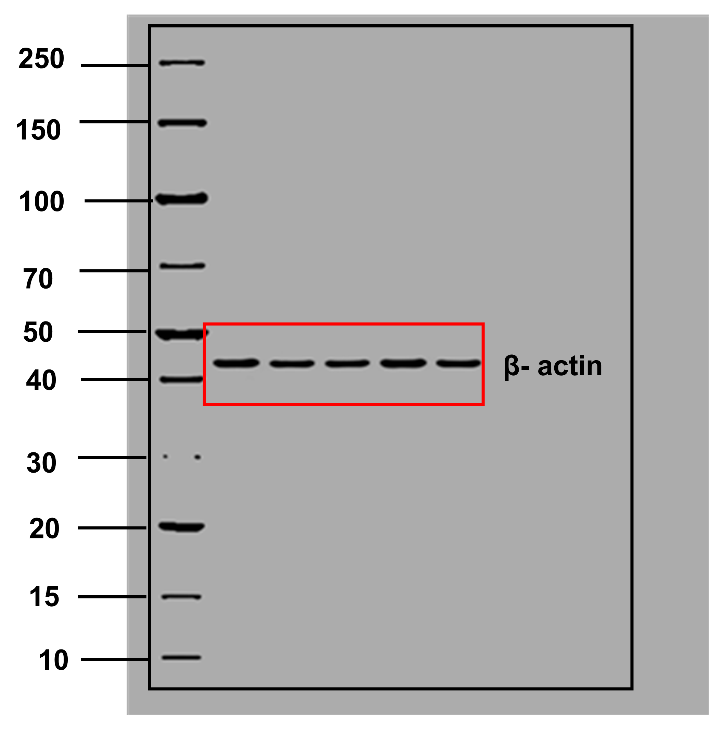

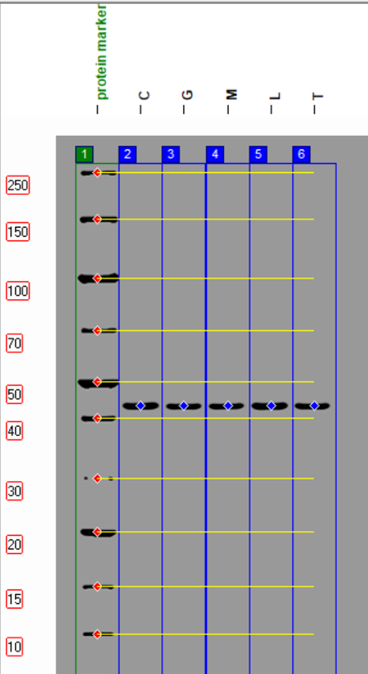

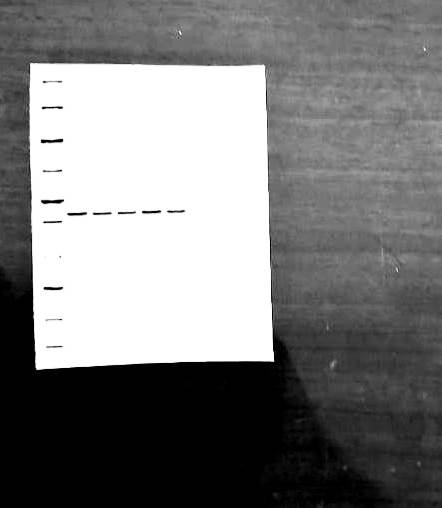


Figure 13: β- actin expression level (1).


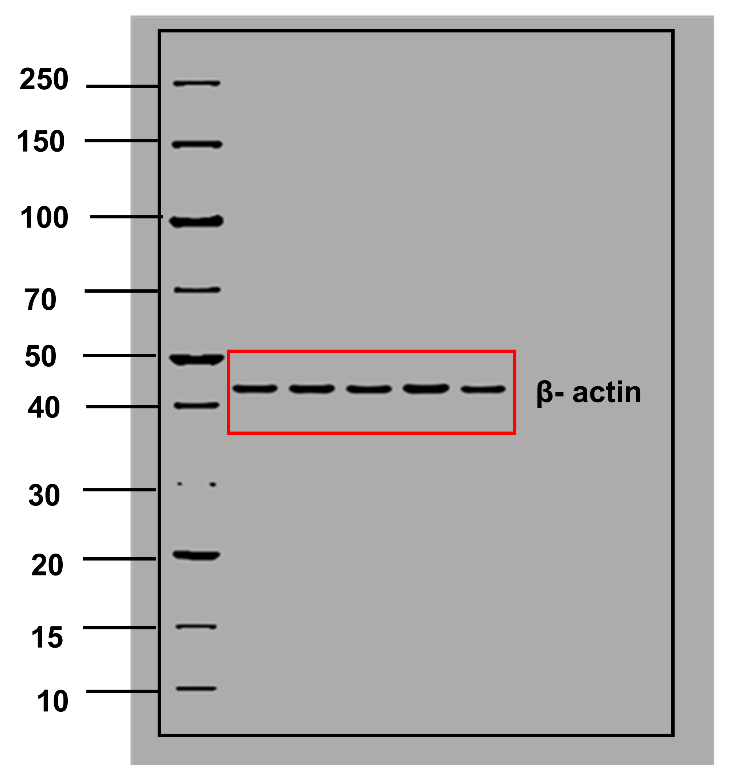

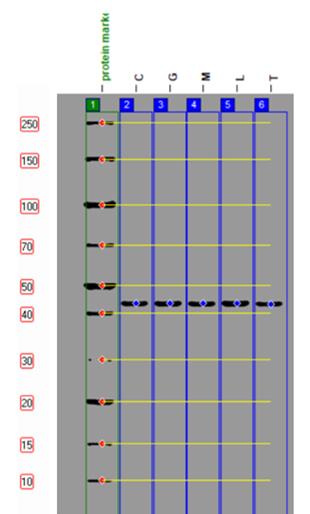

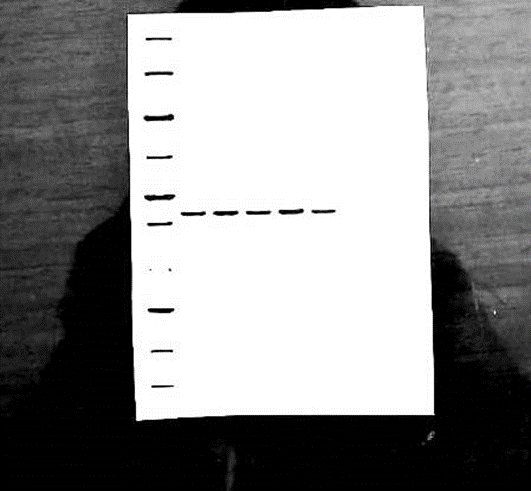


Figure 14: β- actin expression level (2).


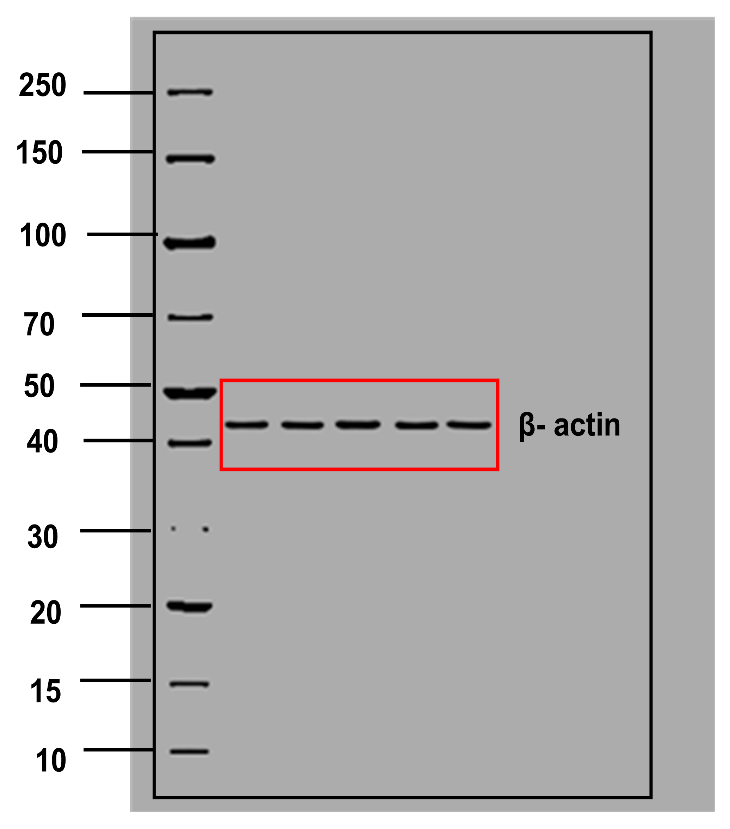

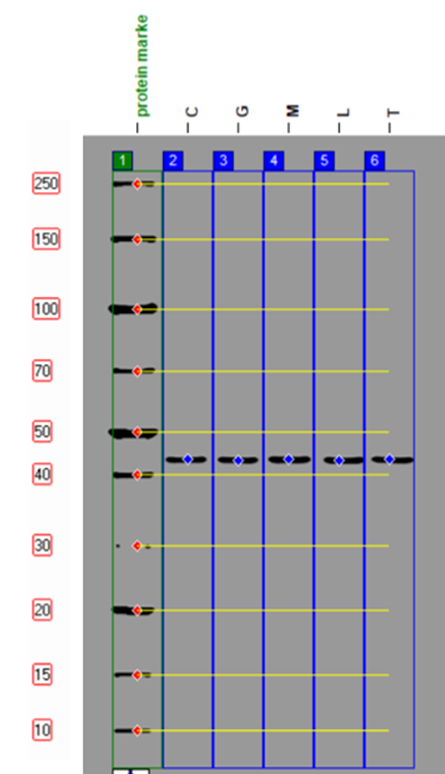


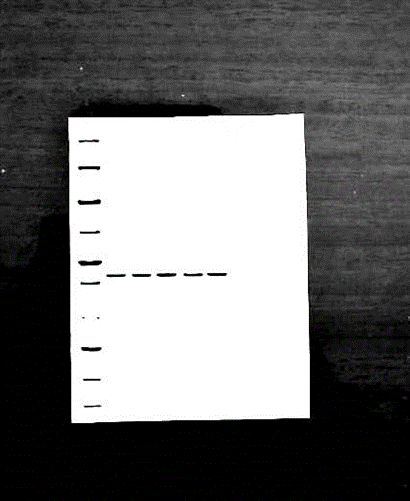


Figure 15: β- actin expression level (3).
